# Human Umbilical Cord Blood Plasma‐Derived Exosomal miR‐410‐3p Alleviates Liver Injury by Regulating the Mitochondria‐Mediated Antiapoptotic Signaling

**DOI:** 10.1002/mco2.70339

**Published:** 2025-08-24

**Authors:** Lin Zhang, Yushuang Ren, Dongsheng Su, Qingyuan Jiang, Huan Peng, Fuyi Cheng, Hantao Zhang, Xue Bai, Xiao Wei, Weixiao Yang, Pusong Zhao, Yixin Ye, Gang Shi, Hongxin Deng

**Affiliations:** ^1^ Department of Biotherapy Cancer Center and State Key Laboratory of Biotherapy West China Hospital, Sichuan University Chengdu China; ^2^ Department of Obstetrics Sichuan Provincial Hospital For Women and Children Chengdu China; ^3^ Department of Clinical Laboratory Sichuan Provincial Hospital For Women and Children Chengdu China; ^4^ West China Biobank West China Hospital, Sichuan University Chengdu China

**Keywords:** apoptosis, exosome, human umbilical cord blood plasma, liver injury, miR‐410‐3p, mitochondria

## Abstract

Severe liver injury is a life‐threatening condition with high mortality and limited therapeutic options. Extensive research on heterochronic parabiosis has highlighted the potent regenerative repair capabilities of young blood in tissue regeneration. However, it remains unclear whether younger blood, specifically umbilical cord blood, can offer similar benefits for tissue repair. In this study, we demonstrate that exosomes derived from umbilical cord blood plasma (CBP‐Exos) exhibit significant therapeutic effects in both acute and chronic liver injury models, outperforming exosomes from young peripheral blood plasma. Treatment with CBP‐Exos notably reduced liver necrosis, lipid peroxidation, and apoptosis in liver tissues of acute liver injury (ALI) mice. Mechanistically, miR‐410‐3p, derived from CBP‐Exos, directly targets the proapoptotic gene Bim for posttranscriptional degradation. The downregulation of Bim facilitates the activation of mitochondrial‐mediated Bcl2‐CytoC antiapoptotic signaling, resulting in the restoration of mitochondrial structure and function, thereby inhibiting hepatocyte apoptosis and oxidative stress. Furthermore, overexpression of miR‐410‐3p significantly improved liver function in ALI mice. These findings identify the therapeutic effects of CBP‐Exos are attributed to the miR‐410‐3p/Bcl2/CytoC axis, laying a foundation for the clinical application of CBP‐Exos and miR‐410‐3p in liver diseases.

## Graphical abstract

(1) CBP‐Exos exhibit better hepatoprotective effects than YBP‐Exos, while OBP‐Exos show no hepatoprotective effects and might even further promote liver injury.

(2) The hepatoprotection effects of CBP‐Exos are attributed to exosomal miR‐410‐3p.

(3) miR‐410‐3p targets Bim and activates the mitochondrial‐mediated Bcl2/CytoC antiapoptotic signaling.

(4) The overexpression of miR‐410‐3p improves liver structure and function in ALI mice.

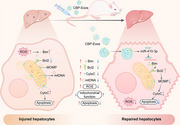

## 1 Introduction

Acute or chronic liver injury can result from various hepatotoxic agents, including viral infections, drug‐induced toxicity, lipid accumulation, and autoimmune responses [1, 2]. It is estimated that more than 1000 drugs currently in use can cause liver injury, of which 353 have been conclusively proven to cause liver injury [3, 4]. Severe liver injury often progresses to liver failure, a condition with limited therapeutic options and a poor prognosis; liver transplantation remains the primary treatment [5]. Consequently, the development of novel therapeutic strategies is urgently needed to address both acute and chronic liver injuries and to avert their progression to liver failure.

Over the past decades, research has indicated that young mouse blood, through heterochronic parabiosis, can restore functionality in aged organs such as the brain, heart, liver, pancreas, kidneys, skeletal muscles, and bones [6, 7]. Subsequent research has focused on identifying the active components (also known as blood‐derived, pro‐youth, or antiaging factors) present in young plasma [8, 9, 10]. Nevertheless, the specific functions of these soluble factors within umbilical cord blood plasma (CBP), an early developmental source, remain largely unexplored. Human umbilical cord blood (UCB), enriched in hematopoietic stem cells, mesenchymal stem cells (MSCs), endothelial progenitor cells, and other stem cells, has been extensively utilized in both basic and clinical research across a range of human diseases [11, 12, 13]. For instance, recent findings indicate that UCB‐derived MSCs have significant therapeutic potential in enhancing cardiac function, managing diabetes, treating rheumatoid arthritis, and particularly in addressing various liver diseases. This potential is primarily due to their paracrine effects, involving the secretion of growth factors, cytokines, and extracellular vesicles (EVs) [14, 15, 16, 17, 18]. Additionally, CBP has been recognized for its regenerative benefits, attributed to its high content of bioactive molecules such as chemokines, neurotrophic factors, and cytokines. These molecules are instrumental in promoting wound healing and play an important role in anti‐inflammation, antiaging, and antiapoptosis and has been widely studied in aging related diseases and brain injury repair [8, 19, 20, 21].

Exosomes, a subset of EVs ranging in diameter from approximately 30 to 150 nm, are secreted by cells and are present in various bodily fluids. They are essential for intercellular communication, facilitating the transfer of microRNAs (miRNAs), mRNAs, proteins, and other molecules to recipient cells [22]. Exosomes derived from serum or plasma represent a rich biological resource with significant potential for therapeutic and diagnostic applications in various diseases [23, 24]. Studies have demonstrated that exosomes from young blood are enriched with bioactive substances, possess tissue repair and regenerative properties, and can be utilized in the treatment of diverse conditions, including liver disorders [25, 26, 27]. This suggests that exosomes derived from younger blood sources, such as umbilical CBP‐derived exosomes (CBP‐Exos), may contain bioactive compounds capable of mediating tissue repair and regeneration. Compared with young blood, UCB offers the advantage of being more readily accessible and devoid of ethical constraints. However, current literature provides limited reports on the therapeutic potential of CBP‐Exos in promoting angiogenesis [28], wound healing [29], and neonatal pulmonary hypertension [30]. Notably, intravenously administered exosomes exhibit a natural tropism for the liver in mice, where they are internalized by intrahepatic cells, establishing a foundation for the application of CBP‐Exos in liver disease treatment. However, the therapeutic promise and function of CBP‐Exos in liver diseases remain underexplored.

miRNAs, small noncoding RNAs approximately 22 nucleotides in length, are widely recognized as essential regulators in various liver diseases, including hepatitis, liver fibrosis, and acute liver injury (ALI). Research has demonstrated that exosomes derived from bone marrow MSCs, enriched with miR‐223‐3p, can mitigate autoimmune hepatitis in mice by suppressing STAT3 and phosphorylated STAT3 (p‐STAT3) expression in LPS‐stimulated macrophages [31]. Another study revealed that exosomal miR‐96‐5p from bone marrow MSCs alleviates nonalcoholic steatohepatitis by modulating the caspase‐2 signaling pathway [32]. Furthermore, in a liver fibrosis mouse model, small EVs originating from tonsil‐derived MSCs were found to inhibit hepatic stellate cell (HSC) activation and reduce liver fibrosis through the action of miR‐486‐5p [33]. Similarly, miR‐150‐5p carried by adipose MSC‐derived EVs suppresses CXCL1 expression, thereby preventing HSC activation and alleviating fibrosis [34]. Additionally, exosomal miR‐455‐3p, derived from human umbilical cord MSCs, was shown to ameliorate interleukin‐6 (IL‐6)‐induced ALI via PI3K signaling pathway activation and promoting hepatocyte proliferation [35].

Herein, we have identified miR‐410‐3p as a crucial functional molecule required for CBP‐Exos‐mediated amelioration of liver injury. The loss‐of‐function of miR‐410‐3p abolished the hepatoprotective effects of CBP‐Exos, indicating its pivotal role in CBP‐Exos‐based liver injury therapy. More interestingly, miR‐410‐3p directly targets the proapoptotic protein Bim for posttranscriptional downregulation, leading to the activation of the mitochondria‐mediated antiapoptotic Bcl2–Cytochrome *c* (CytoC) signaling pathway, thereby exerting a protective effect against hepatocyte damage. Consequently, the miR‐410‐3p/Bim–Bcl2–CytoC axis may represent a key cascade underlying the therapeutic efficacy of CBP‐Exos in liver injury. This study provides new insight into the underlying molecular basis by which CBP‐Exos regulates liver repair through its antiapoptotic properties. Additionally, the effective therapy of miR‐410‐3p in ALI suggests that miR‐410‐3p can be used as a novel miRNA candidate target for the development of nucleic acid molecular drugs for the treatment of severe liver injury.

## 2 Results

### 2.1 The Comparation on the Therapeutic Efficacy of CBP‐Exos, YBP‐Exos, and OBP‐Exos in CCl_4_‐Induced ALI Mice

**FIGURE 1:**
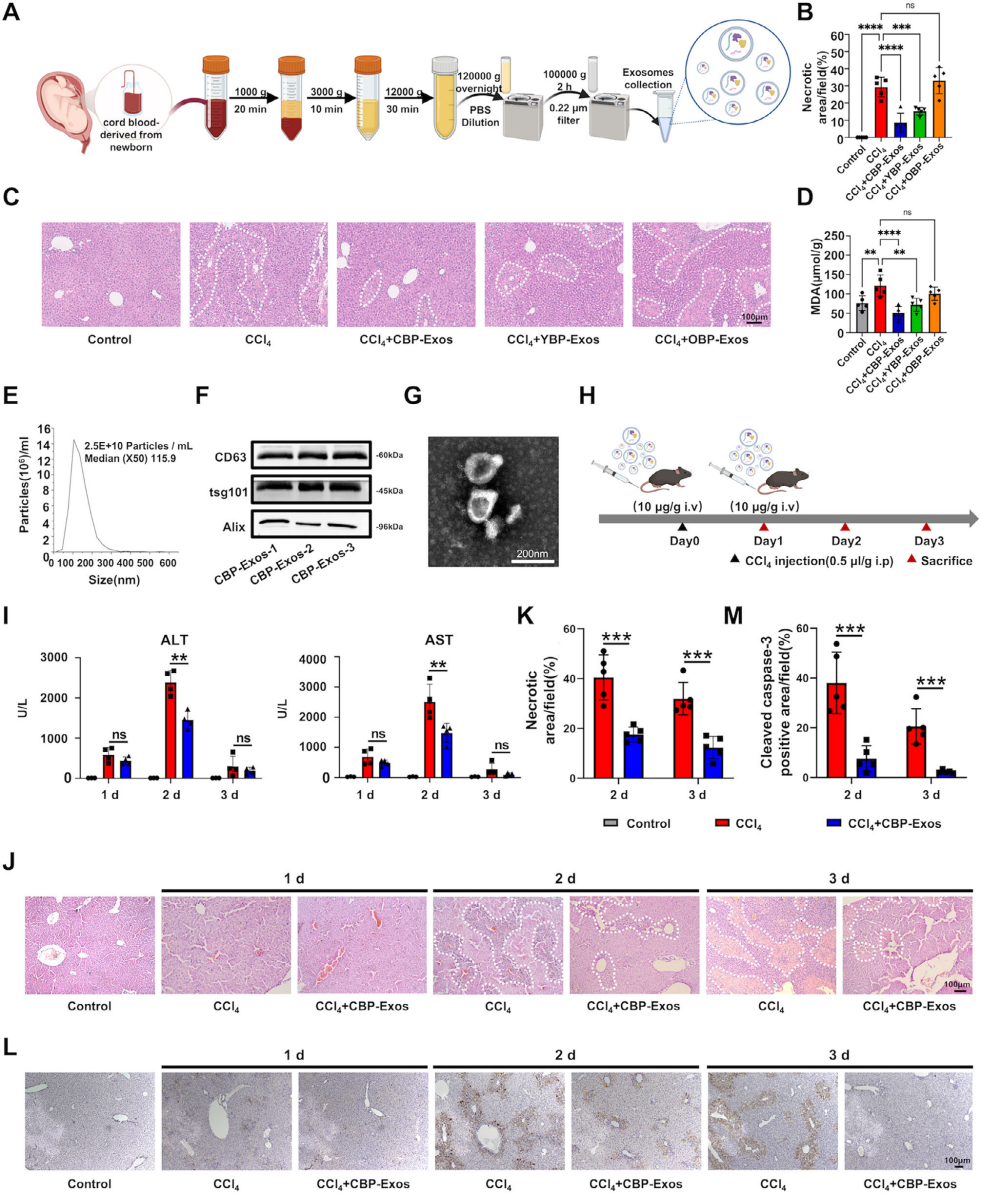
Therapeutic efficacy of exosomes on ALI mice. (A) Schematic diagram of isolation and purification for exosomes. (B and C) H&E staining of liver sections and quantification analysis of the necrotic area of liver tissues in different treatment groups (*n* = 5). (D) Malondialdehyde analysis of liver tissues in different treatment groups (*n* = 5). (E) Size distribution of CBP‐Exos by nanoparticle tracking analysis (NTA)analysis. (F) Exosomal marker identification of CBP‐Exos by Western blot (CBP‐Exos‐1, CBP‐Exos‐2, CBP‐Exos‐3 represented UCB samples from three different healthy donors, respectively). (G) Morphology and size identification of CBP‐Exos by TEM. (H) Schematic illustration of CBP‐Exos‐mediated therapeutic effects on CCl_4_‐induced ALI through antioxidant and antiapoptotic mechanisms. (I) Serum levels of alanine aminotransferase (ALT) and aspartate aminotransferase (AST) after 1 d, 2 d, and 3 d of CBP‐Exos treatment (control group, *n* = 3; CCl_4_ group and CCl_4_+ CBP‐Exos group, *n* = 4). (J and K) H&E staining of liver sections and quantification analysis of the necrotic area of liver tissues after 1 d, 2 d, and 3 d of CBP‐Exos treatment (*n* = 5). (L and M) Immunohistochemistry analysis of cleaved‐caspase‐3 in liver tissues and the positive area quantified by ImageJ after 1 d, 2 d, and 3 d of CBP‐Exos treatment (*n* = 5). In HE staining of the liver, the dotted line indicates the pathological necrosis of the liver and hepatic disruption. In immunohistochemistry analysis of the liver, the dotted line indicates the apoptotic area of the liver. Data are presented as mean ± SEM. One‐way AVONA: ns, not significance; *p < 0.05, **p < 0.01, and ***p < 0.001.

To investigate the effects of CBP‐Exos (22 ≥ years ≤ 27) on liver injury, we initially isolated and characterized CBP‐Exos using an optimized protocol (Figure 1A). As controls, young blood plasma‐derived exosomes (YBP‐Exos, 22 ≥ years ≤ 27) and the old blood plasma‐derived exosomes (OBP‐Exos, 70 ≥ years ≤ 80) were utilized. Next, to determine whether the purified CBP‐Exos, YBP‐Exos, and OBP‐Exos possess the capability to alleviate liver injury, equal amounts of CBP‐Exos, YBP‐Exos, and OBP‐Exos were administered via intravenous injections into CCl_4_‐induced ALI mice respectively. Hematoxylin and eosin (H&E) staining revealed that both CBP‐Exos and YBP‐Exos markedly reduced the necrotic area of liver tissue, while OBP‐Exos appeared to exacerbate liver damage (Figure 1B,C). In addition, malondialdehyde (MDA) levels, a critical byproduct of lipid peroxidation, were significantly reduced in the CBP‐Exos and YBP‐Exos treatment groups (Figure 1D). This finding confirmed that CBP‐Exos and YBP‐Exos treatments effectively mitigated oxidative stress in liver tissue. These findings indicate that CBP‐Exos and YBP‐Exos treatment significantly alleviated ALI, consistent with the previously reported effects of YBP‐Exos in inhibiting liver damage. Importantly, the therapeutic effect of the CBP‐Exos treatment group was better than that of YBP‐Exos treatment group. Next, we will further investigate the function and underlying mechanism of CBP‐Exos in ALI.

### 2.2 CBP‐Exos Alleviate CCl_4_‐Induced ALI Through Antioxidant and Antiapoptotic Effects

Nanoflow cytometry analysis demonstrated that the diameters of CBP‐Exos particles were primarily distributed within the range of 30–150 nm (Figure 1E), a result further validated through transmission electron microscopy (TEM) imaging (Figure S1). Concurrent Western blot analysis and TEM imaging confirmed that CBP‐Exos possessed cup‐ or sphere‐shaped morphology, along with elevated expression levels of exosomal markers such as CD63, tsg101, and Alix (Figure 1F,G). To examine the biodistribution of intravenously transplanted CBP‐Exos in mice, fluorescent labeling using DiR demonstrated that transplanted CBP‐Exos localized predominantly in the liver, with enhanced accumulation in injured liver tissues of ALI‐induced mice (Figure S2A–D). Immunofluorescence staining of liver tissues further demonstrated that more CBP‐Exos were recruited in the liver tissues of injured mice and were mainly colocated with hepatocytes marker ALB (Figure S3A–D). The findings suggest that injured mouse livers exhibit increased recruitment of CBP‐Exos compared with healthy control, with the majority of CBP‐Exos being internalized by hepatocellular parenchymal cells within damaged liver tissues.

To further investigate the regulatory role of CBP‐Exos in ALI, CBP‐Exos were administered intravenously into mice, and their effects on ALI progression were evaluated at various stages (Figure 1H). Notably, CBP‐Exos treatment significantly reduced the serum activities of alanine aminotransferase (ALT) and aspartate aminotransferase (AST) at 2 days postmodeling compared with controls (Figure 1I). Histological analysis using H&E and cleaved‐caspase‐3 immunohistochemistry (IHC) revealed significant improvements in liver tissues following CBP‐Exos administration, including reduced liver necrosis, alleviated hemorrhage, diminished inflammatory infiltration, and decreased hepatocyte apoptosis (Figure 1J–M). To further confirm the therapeutic role of CBP‐Exos in modulating the systemic inflammatory response in ALI, serum analysis showed that the levels of proinflammatory cytokines, including tumor necrosis factor‐alpha (TNF‐α) and IL‐6, were significantly reduced (Figure S4A). Additionally, intrahepatic mononuclear cells were isolated, and the proportions of B cells, CD4+/CD8+ T cells, and neutrophils in CD45+ immune cells were analyzed. The analysis revealed a significant reduction in neutrophil populations, which were elevated in ALI, following CBP‐Exos treatment (Figure S4B–D). Collectively, these findings demonstrate that CBP‐Exos played an important role in hepatoprotection function, including inhibition of hepatocyte apoptosis and oxidative stress.

### 2.3 CBP‐Exos Inhibit Oxidative Stress and Cell Apoptosis in H_2_O_2_‐Induced Hepatocytes

To further explore the antioxidative and antiapoptotic hepatoprotective effects of CBP‐Exos, we conducted in vitro experiments to assess whether CBP‐Exos exert protective effects against oxidative damage in hepatocytes. Primary hepatocytes (PHs) were isolated using in situ liver perfusion, following a previously described protocol [36]. The isolated PHs showed a typical hexagonal morphology with metabolic and storage functions (Figure S5A–D). Cellular uptake of CBP‐Exos was analyzed in AML12 mouse hepatocyte cell lines and isolated PHs. CBP‐Exos were labeled using the lipophilic dye PKH26 and subsequently cocultured with AML12 hepatocytes and PHs, respectively. CBP‐Exos were efficiently internalized by AML12 hepatocytes and PHs, as evidenced by the presence of red fluorescence signals, with no detectable signals observed in control cells treated with dye alone or PBS (Figure S6A,B). Notably, PKH26‐labeled CBP‐Exos were internalized at higher levels in H_2_O_2_‐induced damaged cells compared with normal cells, a finding consistent with the enhanced uptake of CBP‐Exos observed in vivo.

**FIGURE 2:**
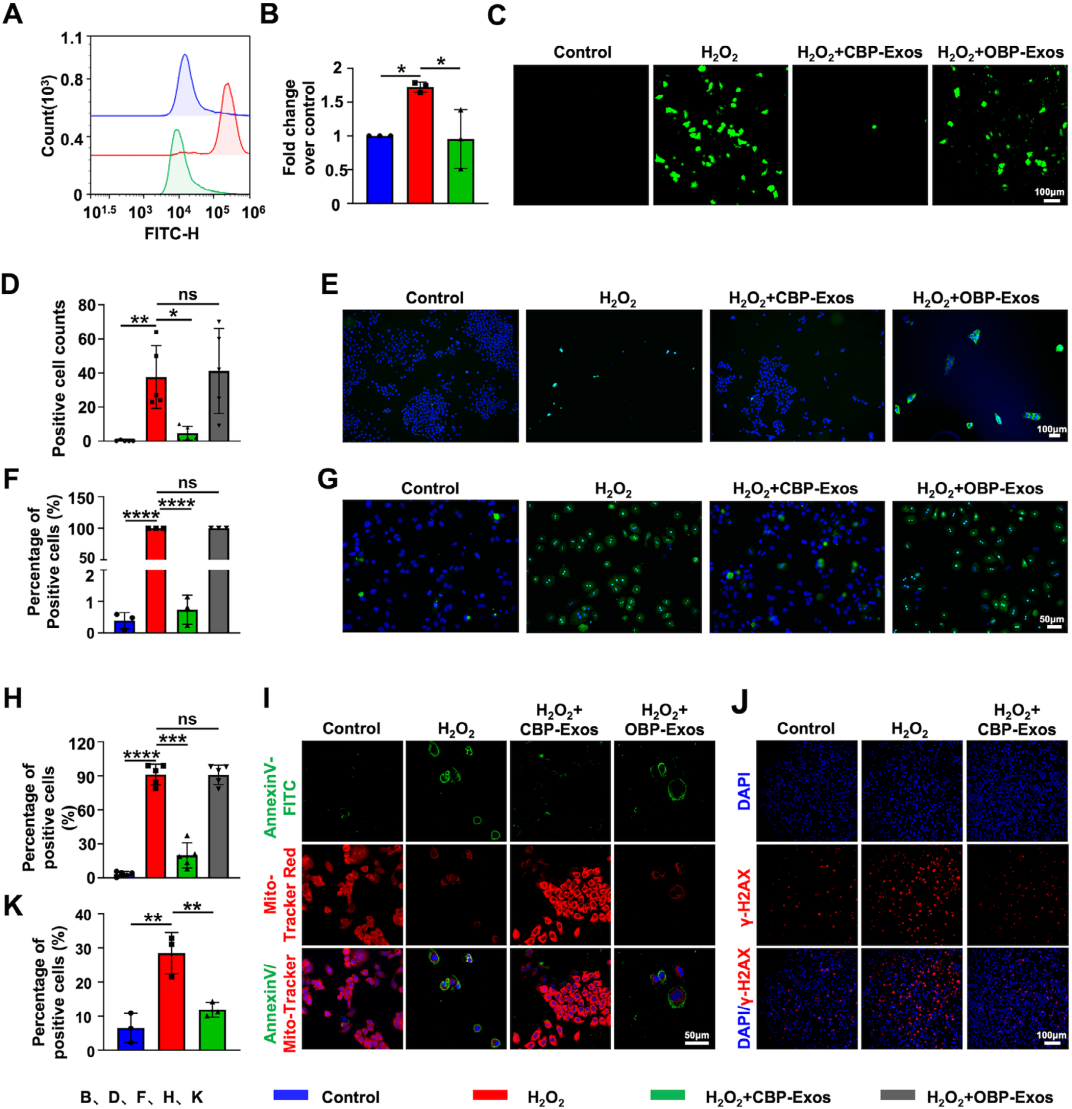
Antioxidative and antiapoptotic effects of CBP‐Exos on H_2_O_2_‐stimulated hepatocytes. (A) Flow analysis of ROS level in AML12 hepatocytes treated with CBP‐Exos. (B) Quantification of the ratio of ROS level in each group to the negative control group (*n* = 3). (C) Immunofluorescence analysis of ROS‐positive cells in PHs treated with CBP‐Exos or OBP‐Exos, respectively. (D) Quantification of the ROS‐positive cells in each group (*n* = 5). (E) The TUNEL staining images of AML12 hepatocytes treated with CBP‐Exos or OBP‐Exos, respectively. (F) Quantitative analysis of the TUNEL staining images (*n* = 3). (G) The TUNEL staining images of PHs treated with CBP‐Exos or OBP‐Exos, respectively. (H) Quantitative analysis of the TUNEL staining images (*n* = 4). (I) Mitochondrial membrane potential and apoptosis staining images of AML12 hepatocytes treated with CBP‐Exos or OBP‐Exos, respectively. (J) Immunofluorescence analysis of γ‐H2AX in AML12 hepatocytes treated with CBP‐Exos. (K) Quantification of the percentage of γ‐H2AX positive AML12 hepatocytes (*n* = 3). Data are presented as mean ± SEM. One‐way AVONA: ns, not significance; *p < 0.05, **p < 0.01, ***p < 0.001, and ****p < 0.0001.

Next, hepatocytes were pretreated with CBP‐Exos and then stimulated with H_2_O_2_ to induce oxidative stress, simulating the liver injury model caused by CCl_4_ in vivo. As expected, reactive oxygen species (ROS) levels were elevated in AML12 hepatocytes treated with H_2_O_2_, while ROS levels were significantly reduced following pretreatment with CBP‐Exos (Figure 2A,B). Subsequently, the antioxidative effects of CBP‐Exos were further investigated in PHs, with OBP‐Exos included as a control. ROS levels were markedly reduced in PHs pretreated with CBP‐Exos, whereas OBP‐Exos provided minimal protective effects (Figure 2C,D). Given the observed reduction in apoptosis in liver tissues treated with CBP‐Exos in CCl_4_‐induced ALI, we next evaluated the antiapoptotic effects in vitro. Pretreatment with CBP‐Exos significantly reduced apoptotic cell numbers in both AML12 hepatocytes and PHs compared with the H_2_O_2_‐stimulated group, whereas OBP‐Exos had no significant impact, highlighting the pronounced antiapoptotic effect of CBP‐Exos (Figure 2E–H). Cytocalcein, Annexin V, and 7‐AAD triple staining assays were employed to quantify apoptotic cell populations, highlighting viable cells (Cytocalcein‐positive), early apoptotic cells (Annexin V‐positive), and necrotic or late apoptotic cells (7‐AAD‐positive) [37]. As expected, immunofluorescence staining confirmed that CBP‐Exos treatment significantly reduced the number of Annexin V‐positive and Annexin V/7‐AAD double‐positive apoptotic cells (Figure S7). Since mitochondria play a critical role in regulating apoptosis [38], we evaluated the impact of CBP‐Exos on mitochondrial function. Reduced mitochondrial membrane potential, reflected by decreased MitoTracker Red fluorescence, indicated mitochondrial depolarization during ROS‐induced damage, a process reversed by CBP‐Exos pretreatment, which additionally inhibited apoptosis (Figures 2I and S8). In addition to mitochondrial dysfunction, oxidative stress caused by elevated ROS levels also induces DNA damage, which accelerates apoptosis. Immunofluorescence staining of the DNA damage marker γ‐H2AX demonstrated that CBP‐Exos treatment substantially reduced γ‐H2AX expression induced by H_2_O_2_ (Figure 2J,K). Taken together, the above results indicated that CBP‐Exos exerts a hepatoprotective effect by reducing ROS levels, preventing DNA damage, restoring mitochondrial function, and inhibiting apoptosis in H_2_O_2_‐induced hepatocytes.

### 2.4 miR‐410‐3p is a Key Component in CBP‐Exos‐Mediated Hepatoprotection

**FIGURE 3:**
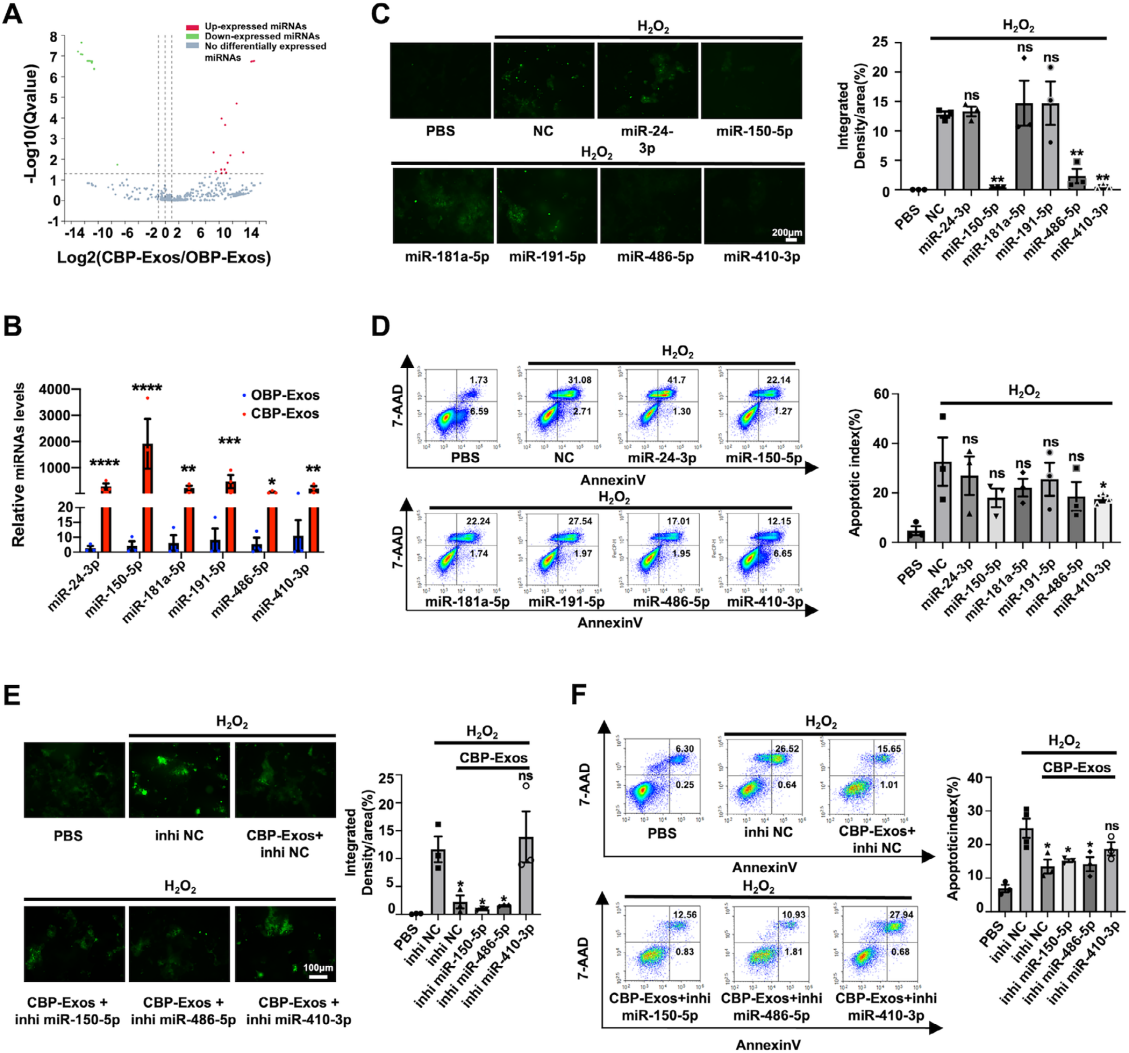
The hepatoprotection function of miR‐410‐3p on H_2_O_2_‐stimulated AML12 hepatocytes. (A) Volcano plot analysis of differential miRNAs between CBP‐Exos and OBP‐Exos. (B) RT‐qPCR analysis of the six differentially expressed miRNAs in CBP‐Exos and OBP‐Exos. Data are normalized to spiked cel‐miR‐39 (*n* = 3). (C) Immunofluorescence analysis of ROS‐positive cells in AML12 hepatocytes transfected with a mimic of indicated miRNA or negative control (NC) for 24 h followed by H_2_O_2_‐stimulated hepatocytes (*n* = 3). (D) Flow cytometric analysis of apoptosis of AML12 hepatocytes transfected with a mimic of indicated miRNA or negative control (NC) for 24 h followed by H_2_O_2_‐stimulated hepatocytes (*n* = 3). (E) Immunofluorescence analysis of ROS‐positive cells in AML12 hepatocytes preincubated with CBP‐Exos and transfected with an inhibitor of indicated miRNA or negative control (inhiNC) for 24 h followed by H_2_O_2_‐stimulated hepatocytes (*n* = 3). (F) Flow cytometric analysis of apoptosis of AML12 hepatocytes preincubated with CBP‐Exos and transfected with an inhibitor of indicated miRNA or negative control (inhiNC) for 24 h followed by H_2_O_2_‐stimulated hepatocytes (*n* = 3). Data are presented as mean ± SEM. One‐way AVONA: ns, not significance; *p < 0.05, **p < 0.01, ***p < 0.001, and ****p < 0.0001.

Previous studies have demonstrated that EVs transport miRNAs, which serve as crucial regulators in EV‐mediated intercellular communication during liver injury [39]. Consequently, we hypothesized that specific miRNAs might play a role in CBP‐Exos‐mediated hepatoprotection. To test this hypothesis, a miRNA profiling assay was conducted to compare CBP‐Exos and OBP‐Exos using MGI2000 high‐throughput sequencing. Thirty‐four miRNAs with significant differential expression (fold change > 2.0; *p* < 0.05) were identified, of which 16 were upregulated in CBP‐Exos relative to OBP‐Exos (Figure 3A). Potential miRNAs were further screened from these 16 candidates based on two criteria: (1) homology between human and mouse miRNAs, and (2) data points in the upper‐right quadrant of the volcano plot. Accordingly, miR‐410‐3p was identified. Based on these criteria, miR‐410‐3p was identified as a candidate. Given the hepatoprotective effects of CBP‐Exos, a miRNA–mRNA regulatory network analysis was performed to investigate the apoptosis‐related pathways in liver injury, focusing on differentially expressed miRNAs and their target genes. This analysis identified miR‐24‐3p (fold change > 2.0; *p* < 0.05) as another candidate potentially involved in oxidative stress and apoptosis signaling pathways (Figure S9A). Additionally, previous studies have highlighted that the high enrichment of functional molecules in exosomes is crucial for their biological activity. This led to the identification of the top 10 miRNAs (fold change > 2.0; *p* < 0.05) with the highest expression levels in CBP‐Exos. Notably, miR‐150‐5p, miR‐181a‐5p, miR‐191‐5p, and miR‐486‐5p were implicated in liver injury‐related signaling pathways and exhibited homology between humans and mice (Figure S9B).

The screened six differentially expressed miRNAs were further validated through RT‐qPCR analysis, revealing that all were upregulated in CBP‐Exos compared with OBP‐Exos (Figure 3B). miRNA mimics, which are known to enhance cellular miRNA abundance, were utilized to investigate gain‐of‐function effects. RT‐qPCR analysis following transfection of corresponding miRNA mimics showed a robust upregulation of the six miRNAs in both AML12 hepatocytes and PHs (Figure S10A,B). Functional effects of these six miRNAs were assessed using miRNA mimics in an in vitro ALI model. miR‐150‐5p, miR‐486‐5p, and miR‐410‐3p emerged as three key candidates due to their ability to significantly restore hepatocyte morphology, reduce ROS levels, and suppress apoptosis in H_2_O_2_‐treated hepatocytes (Figures S10C,D, 3C,D, and S11A,B). To determine the dominant miRNAs responsible for CBP‐Exos‐mediated hepatoprotection, miRNA inhibitors were employed to evaluate the functional contributions of specific miRNAs. Notably, only the miR‐410‐3p inhibitor group exhibited elevated ROS levels and apoptosis, whereas miR‐150‐5p and miR‐486‐5p inhibitors showed no such effects, highlighting miR‐410‐3p as the primary contributor to CBP‐Exos‐mediated protective effects in H_2_O_2_‐stimulated hepatocytes (Figures 3E,F and S11C,D). To further validate that miR‐410‐3p inhibits hepatocyte apoptosis induced by oxidative stress, we used cleaved‐caspase‐3 immunofluorescence staining to evaluate the activation status of caspase proteins in hepatocytes. The results demonstrated that miR‐410‐3p treatment significantly reduced hepatocyte apoptosis under H_2_O_2_ exposure (Figure S13). Additionally, CBP‐Exos treatment showed similar effects, consistent with the previously presented TUNEL staining results (Figure S12). These findings further confirm that miR‐410‐3p mediates antiapoptotic effects, providing additional evidence to support the conclusions of this study. Notably, exosome degradation analysis employing ribonuclease (RNase), proteinase K, and Triton X‐100 revealed that miR‐410‐3p was predominantly localized within intact exosomes (Figure S13). Altogether, these findings suggest the essential role of miR‐410‐3p in CBP‐Exos‐mediated alleviation of oxidative stress and apoptosis in hepatocytes.

### 2.5 miR‐410‐3p Exerts Antiapoptotic Effects via Targeting Bim in Hepatocytes

**FIGURE 4:**
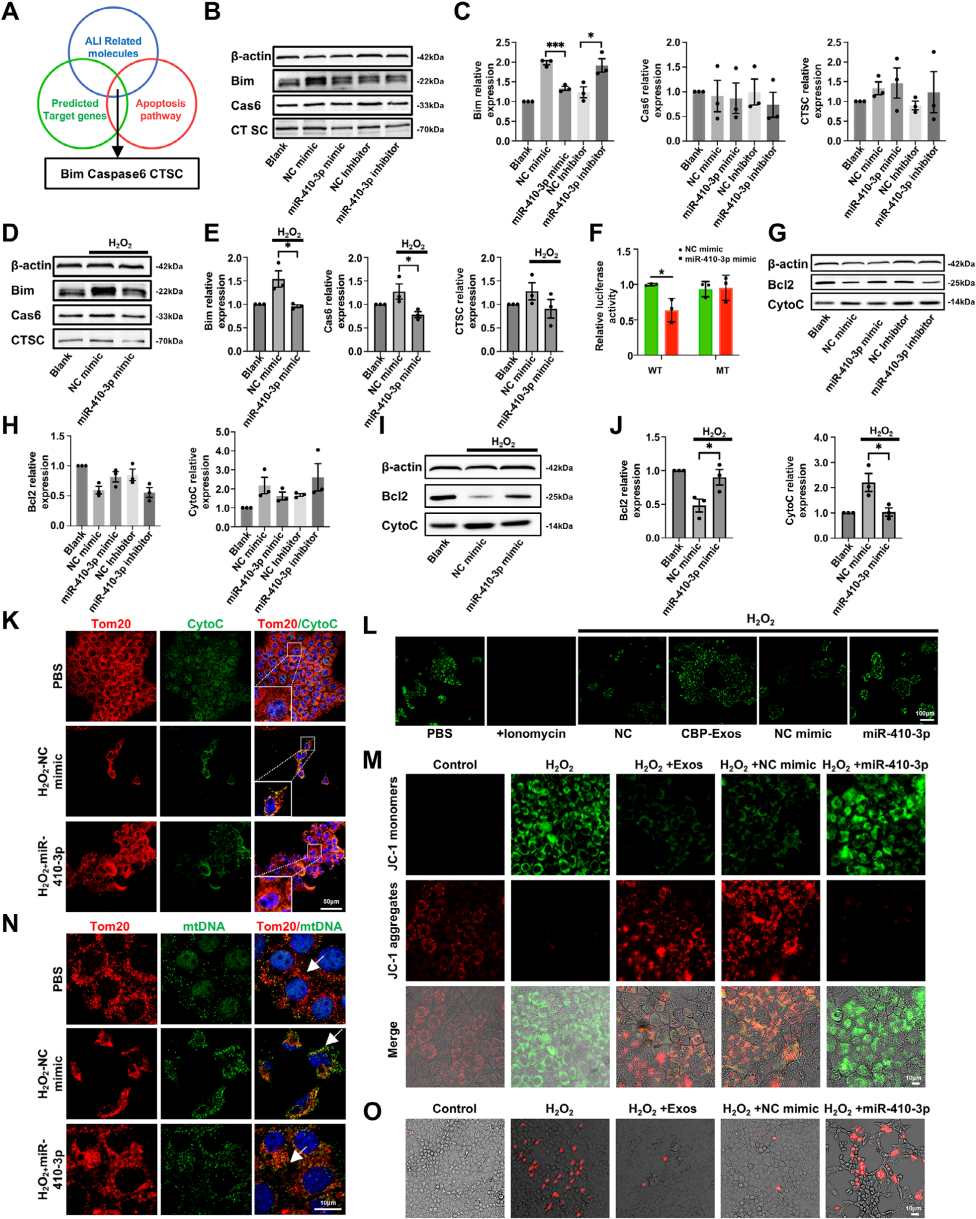
miR‐410‐3p targets Bim and regulates mitochondria‐mediated antiapoptotic Bcl2–CytoC signaling in hepatocytes. (A) Screening scheme for putative target genes that might contribute to the antiapoptotic effects of miR‐410‐3p. (B and C) Western blot analysis showing protein expressions of the three putative miR‐410‐5p target genes in PHs treated with miR‐410‐3p mimic, mimic negative control (NC mimic), miR‐410‐3p inhibitor, or inhibitor NC. (D and E) Western blot analysis showing protein expressions of the three putative miR‐410‐5p target genes in H_2_O_2_‐stimulated PHs treated with miR‐410‐3p mimic or mimic negative control (NC mimic). (F) Luciferase assays of 293T cells cotransfected with miR‐410‐3p mimic or mimic NC and reporter plasmids containing 3′UTR wild type or mutated miR‐410‐3p binding sites for the Bim target gene (*n* = 3). (G and H) Western blot analysis showing protein expressions of Bcl2 and CytoC in PHs treated with miR‐410‐3p mimic, mimic negative control (NC mimic), miR‐410‐3p inhibitor, or inhibitor NC. (I and J) Western blot analysis showing protein expressions of Bcl2 and CytoC in H_2_O_2_‐stimulated PHs treated with miR‐410‐3p mimic or mimic negative control (NC mimic). (K) Representative immunofluorescence images of Tom20 (red) and CytoC (green) in AML12 hepatocytes in H_2_O_2_‐stimulated AML12 hepatocytes treated with miR‐410‐3p mimic or mimic negative control (NC mimic). (L) Representative immunofluorescence images of the opening of the mitochondrial permeability transition pores (mPTP) in H_2_O_2_‐stimulated AML12 hepatocytes treated with CBP‐Exos, negative control (NC), miR‐410‐3p mimic, or mimic negative control (NC mimic). (M) Representative immunofluorescence images of the JC‐1 stanning in H_2_O_2_‐stimulated AML12 hepatocytes treated with CBP‐Exos, negative control (NC), miR‐410‐3p mimic, or mimic negative control (NC mimic). (N) Representative immunofluorescence images of Tom20 (red) and mtDNA (green) and the white arrowheads indicate the leakage of mtDNA in H_2_O_2_‐stimulated AML12 hepatocytes treated with miR‐410‐3p mimic or mimic negative control (NC mimic). (O) Representative immunofluorescence images of the MitoROS stanning in H_2_O_2_‐stimulated AML12 hepatocytes treated with CBP‐Exos, negative control (NC), miR‐410‐3p mimic, or mimic negative control (NC mimic).

To elucidate the potential cell‐protective mechanisms underlying miR‐410‐3p activity, target genes of mmu‐miR‐410‐3p were predicted using TargetScan, MiRanda, and PITA databases and functionally categorized using the DAVID gene functional classification tool. Among the possible miR‐410‐3p target genes, we finally focused on Bim, caspase‐6, and CTSC, which are involved in both ALI and the apoptosis pathway (Figure 4A). We found that the miR‐410‐3p mimic significantly decreased Bim protein levels, while the miR‐410‐3p inhibitor increased Bim protein levels in hepatocytes, and no significant change of caspase‐6 and CTSC expression was observed (Figures 4B,C and S14A). Additionally, we further identified the target gene of miR‐410‐3p in hepatocytes subjected to oxidative stress. The results revealed a significant increase in the protein levels of Bim and caspase‐6 following H_2_O_2_ stimulation, whereas treatment with the miR‐410‐3p mimic markedly reduced the protein levels of both Bim and caspase‐6 (Figures 4D,E and S14B).

Caspase‐6, a member of the cysteine protease family, has been reported to participate in various cell death regulatory pathways in response to damage‐associated molecular patterns or pathogen‐associated molecular patterns and is critical for initiating apoptosis [40, 41]. This could explain the observed reduction in caspase‐6 protein expression following miR‐410‐3p mimic treatment in H_2_O_2_‐stimulated hepatocytes, but not in unstimulated hepatocytes, suggesting that caspase‐6 might function downstream of Bim. These findings raised a possibility that Bim could serve as a downstream effector protein in mediating miR‐410‐3p‐driven hepatoprotection. To test this hypothesis, potential miR‐410‐3p binding sites within the 3′‐untranslated regions (3′UTRs) of Bim were analyzed through luciferase reporter assays. Wild‐type and mutant Bim 3′UTRs, containing putative miR‐410‐3p binding sites, were cloned into reporter plasmids, and their responsiveness to miR‐410‐3p was assessed in 293T cells. As expected, miR‐410‐3p significantly decreased luciferase activity in the wild‐type Bim 3′UTR construct, whereas no effect was observed in constructs with mutated miR‐410‐3p binding sites (Figure 4D). Altogether, these findings demonstrate that miR‐410‐3p mediates antiapoptotic effects by directly targeting Bim in injured hepatocytes.

### 2.6 miR‐410‐3p Activates the Mitochondria‐Mediated Bcl2–CytoC Antiapoptotic Signaling Pathway

To clarify the precise mechanism by which Bim, a direct target of miR‐410‐3p, contributes to antiapoptotic signaling, we investigated the downstream molecules regulated by Bim that are involved in the antiapoptotic signaling pathway. Cellular stress or injury signals typically activate proapoptotic BH3‐only proteins, including Bim and PUMA, which are sequestered by prosurvival proteins, such as Bcl‐2 and Bcl‐xl, under normal conditions. However, when these prosurvival proteins are depleted or unable to sequester proapoptotic activators, proapoptotic proteins induce mitochondrial outer membrane permeabilization, leading to the release of apoptogenic molecules, including Cytochrome *c* (CytoC), which subsequently activates the classical caspase signaling cascade, initiating apoptosis [42]. Accordingly, we hypothesized that miR‐410‐3p might promote antiapoptotic signaling by targeting Bim for posttranscriptional degradation. To test this hypothesis, immunoblot analyses were conducted to assess the impact of miR‐410‐3p on the expression levels of the prosurvival protein Bcl‐2 and the apoptogenic molecule CytoC, both of which are key downstream effectors of Bim. Notably, miR‐410‐3p mimic led to a marked increase in Bcl2 expression and a decrease in CytoC, whereas miR‐410‐3p inhibitor appears to downregulate the expression of Bcl2 and upregulate the expression of CytoC (Figures 4G,H and S14C). Consistently, miR‐410‐3p mimic remarkably upregulated the expression of Bcl2 and decreased the expression of CytoC in H_2_O_2_‐stimulated oxidative damaged hepatocytes (Figures 4I,J and S14D).

CytoC, a key apoptogenic molecule, is known to interact with Apaf‐1 and caspase‐9 to form apoptosomes, which subsequently activate caspase‐3, caspase‐6, and caspase‐7, thereby initiating mitochondria‐mediated apoptosis [38]. To further investigate the role of CytoC following miR‐410‐3p treatment in H_2_O_2_‐stimulated hepatocytes, immunofluorescence staining assays were employed to examine CytoC expression and release. CytoC, visualized using green fluorescence, was colocalized with TOM20 (red fluorescence) and exhibited a weak, evenly distributed pattern in unstimulated AML12 hepatocytes. ROS‐triggered TOM20‐labeled mitochondria were disordered and crumpled into clumps, and the green fluorescence intensity of labeled CytoC was significantly increased, while miR‐410‐3p mimic restored mitochondrial morphology and reversed the increase and release of CytoC in AML12 hepatocytes exposed to H_2_O_2_ (Figure 4K). Meanwhile, further examination of the openness of mitochondrial permeability transition pore (MPTP) demonstrated that compared with the NC group, both CBP‐Exos and miR‐410‐3p mimic dramatically decreased the openness of MPTP, indicating that ROS‐triggered increase of CytoC release might attribute to the increase openness of MPTP (Figure 4L). To dynamically assess the impact of CBP‐Exos or miR‐410‐3p mimic on mitochondrial membrane potential, we utilized JC‐1 staining. Consistent with the MPTP results, the results demonstrated that CBP‐Exos or miR‐410‐3p mimic treatment significantly maintained the mitochondrial membrane potential in H_2_O_2_‐stimulated hepatocytes (Figure 4M). Apart from the aggravated release of proapoptotic molecules caused by the increase of mitochondrial MPTP openness, the release of proinflammatory molecules such as mitochondrial DNA (mtDNA) also increase to thus aggravate cell damage. ROS‐triggered cytosolic mtDNA leakage, as indicated by enlarged mtDNA clusters without TOM20 colocalization (white arrows), in AML12 hepatocytes was reduced by the miR‐410‐3p mimic treatment (Figure 4N). To further investigate the mitochondrial oxidative stress status, we performed MitoROS assays. Consistent with these findings, MitoROS levels were significantly reduced following CBP‐Exos or miR‐410‐3p mimic treatment compared with the control group (Figure 4O). These results underscore the ability of CBP‐Exos and miR‐410‐3p to mitigate oxidative stress at the mitochondrial level.

**FIGURE 5:**
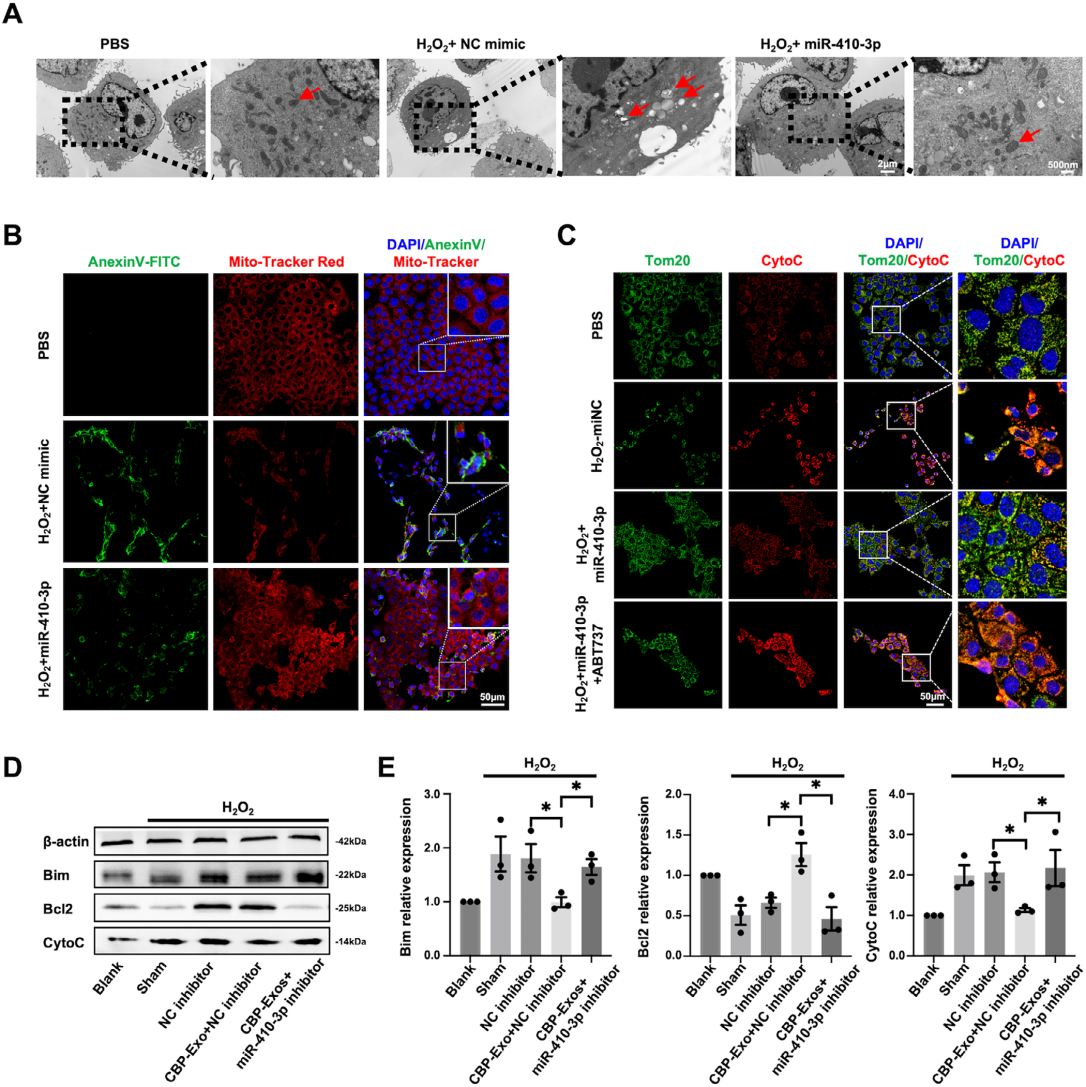
miR‐410‐3p restores mitochondrial structure and function in hepatocytes. (A) Representative TEM images of mitochondria in H_2_O_2_‐stimulated AML12 hepatocytes treated with miR‐410‐3p mimic or mimic negative control (NC mimic). (B) Representative immunofluorescence images of MitoTracker (red) and AnnexinV (green) in H_2_O_2_‐stimulated AML12 hepatocytes treated with miR‐410‐3p mimic or mimic negative control (NC mimic). (C) Represented immunofluorescence images of Tom20 (green) and CytoC (red) in H_2_O_2_‐stimulated AML12 hepatocytes treated with Bcl2 antagonist, ABT737 and transfected with miR‐410‐3p mimic or mimic negative control (NC). (D and E) Western blot analysis showing Bim, Bcl2, and CytoC expression in PHs preincubated with CBP‐Exos and transfected with miR‐410‐3p inhibitor, or inhibitor NC.

Subsequent investigations into mitochondrial structure and function revealed that miR‐410‐3p mimic treatment effectively alleviated mitochondrial swelling, fragmentation, cristae damage, and deformation, restored mitochondrial membrane potential, and suppressed apoptosis in H_2_O_2_‐stimulated hepatocytes (Figure 5A,B). These findings suggest that miR‐410‐3p may regulate mitochondrial‐mediated Bcl2/CytoC antiapoptotic signaling pathways. To further investigate whether the hepatoprotective effects of miR‐410‐3p are exerted through Bim‐mediated regulation of mitochondrial Bcl2–CytoC signaling, the Bcl2 antagonist ABT737 was employed to block Bcl2/CytoC signaling in H_2_O_2_‐stimulated hepatocytes. The results showed that miR‐410‐3p mimic failed to reduce CytoC expression levels in damaged hepatocytes and was unable to restore mitochondrial structure or function when Bcl2/CytoC signaling was inhibited (Figure 5C). In addition, to prove that miR‐410‐3p is required for CBP‐Exos‐mediated activation of Bim/Bcl2/CytoC antiapoptotic signaling. Immunoblot analysis demonstrated that increases in the levels of Bim and CytoC and decreases in the levels of Bcl2 in PHs treated with H_2_O_2_ were reversed by CBP‐Exos, but this beneficial effect was abolished following transfection with miR‐410‐3p inhibitor (Figure 5D,E). In conclusion, these findings demonstrate that miR‐410‐3p, a functional component of CBP‐Exos, plays a hepatoprotection role against H_2_O_2_‐induced oxidative damage by activating antiapoptotic signaling pathways mediated through Bim–Bcl2–CytoC mitochondrial mechanisms.

### 2.7 miR‐410‐3p Alleviates CCl_4_‐Induced ALI Through Activating Mitochondrial‐Mediated Antiapoptotic Signals

**FIGURE 6:**
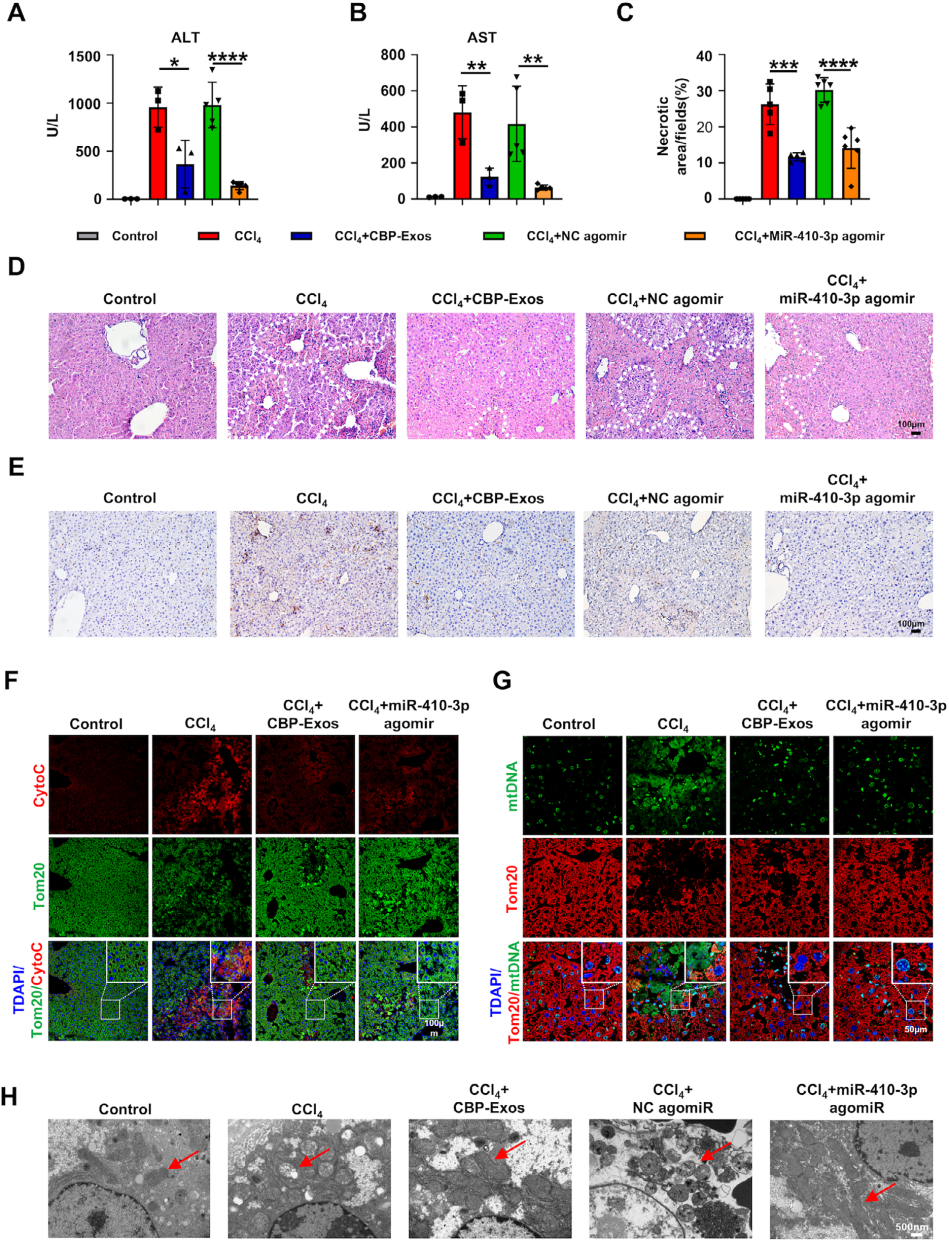
miR‐410‐3p agomir alleviates CCl_4_‐induced acute liver injury. (A and B) Serum levels of ALT and AST after 2 d of miR‐410‐3p agomir treatment (control group, CCl_4_ group, and CCl_4_+ CBP‐Exos group, *n* = 3; NC agomir group and miR‐410‐3p agomir group, *n* = 5). (C and D) H&E staining of liver sections and quantification analysis of necrotic area of liver tissues in different treatment groups (*n* = 5). In HE staining of the liver, the dotted line indicates the pathological necrosis of the liver and hepatic disruption. (E) Immunohistochemistry analysis of F4/80 of liver tissues in different treatment groups (*n* = 5). (F) Representative micrographs of Tom20 (green) and CytoC (red) costaining in mouse liver. (G) Representative micrographs of Tom20 (red) and CytoC (green) costaining in mouse liver. (H) Representative TEM images of mitochondria in different treatment groups.

The hepatoprotective effects of miR‐410‐3p observed in H_2_O_2_‐stimulated hepatocytes prompted further investigation into its therapeutic potential in vivo. For this purpose, miR‐410‐3p agomir was synthesized to assess its therapeutic efficacy in ALI mice model. Compared with the control group, mice treated with miR‐410‐3p agomir exhibited significantly reduced serum levels of ALT and AST (Figure 6A,B). H&E staining revealed that miR‐410‐3p agomir administration, similar to CBP‐Exos, significantly decreased the extent of hepatocellular necrosis (Figure 6C,D). Immunohistochemical staining for F4/80 revealed a significant reduction in macrophage recruitment in liver tissues from the miR‐410‐3p agomir‐treated group (Figure 6E). Furthermore, to evaluate whether miR‐410‐3p agomir confers hepatoprotection via mitochondrial‐mediated CytoC signaling in oxidative damaged mouse livers, immunofluorescent staining was performed to examine CytoC expression and mtDNA leakage. The analysis revealed that miR‐410‐3p agomir, like CBP‐Exos, significantly suppressed CytoC expression and mtDNA release in CCl_4_‐treated liver tissues. Notably, mitochondrial distribution, visualized using TOM20 labeling, was also restored (Figure 6F,G). As anticipated, both CBP‐Exos and miR‐410‐3p agomir treatments ameliorated mitochondrial damage by reducing mitochondrial swelling, fragmentation, cristae deformation, and restoring mitochondrial morphology and structure (Figure 6H). Overall, our results suggest that miR‐410‐3p exhibits therapeutic effects comparable to CBP‐Exos by improving liver structure and function, with its mechanism partially attributed to mitochondrial‐mediated CytoC antiapoptotic signaling pathways.

To further validate the broad applicability of CBP‐Exos and miR‐410‐3p in other models of ALI, we established an acetaminophen (APAP)‐induced ALI model. APAP overdose is a well‐recognized cause of drug‐induced liver injury and represents a mechanistically distinct model compared with the CCl_4_‐induced liver injury model. In the APAP‐induced liver injury model, both CBP‐Exos and miR‐410‐3p treatment demonstrated significant therapeutic effects. Specifically, CBP‐Exos and miR‐410‐3p reduced serum ALT and AST levels, alleviated hepatocyte necrosis and inflammatory infiltration, and preserved tissue architectural integrity (Figure S15A,B). These findings are consistent with the therapeutic effects observed in CCl_4_‐induced liver injury, suggesting that CBP‐Exos and miR‐410‐3p exhibit robust hepatoprotective effects across different liver injury models.

### 2.8 CBP‐Exos Inhibits the Progression of Liver Fibrosis in CCl_4_‐Induced Chronic Liver Injury

**FIGURE 7:**
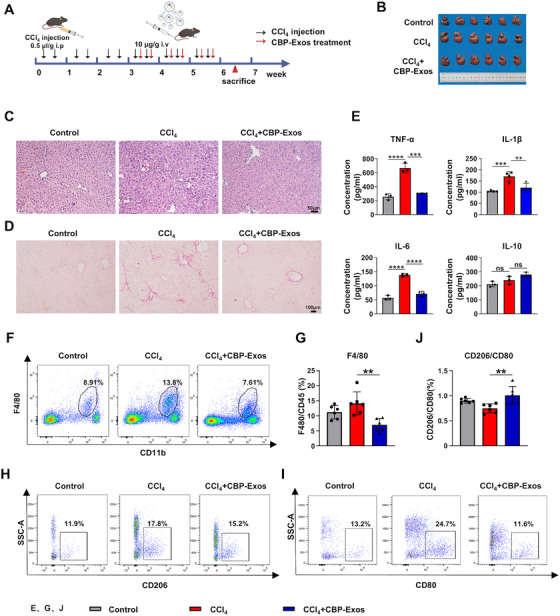
CBP‐Exos alleviates CCl_4_‐induced chronic liver injury. (A) Schematic illustration of the CBP‐Exos for CCl_4_‐induced liver fibrosis (LF) therapy. (B) The general appearance of the livers. (C) H&E staining of liver sections. (D) Sirius red (SR) staining of liver sections. (E) Serum inflammatory cytokines (TNF‐α, IL‐6, IL‐1β, and IL‐10) were determined by Elisa Detection Kit (*n* = 3 or 4). (F and G) Flow cytometry analyses of F4/80 cells in intrahepatic CD45+ immune cells of each treatment group. Statistical analyses of the percent of F4/80 cells in each group. Data are presented as the means ± SD (*n* = 6 mice per group). (H and I) Flow cytometry analyses of CD206+ and CD80+ cells in intrahepatic F4/80‐positive mononuclear cells of each treatment group. (J) Statistical analyses of the ratio of CD206+/CD80+ cells in each group. Data are presented as the means ± SD (*n* = 6 mice per group).

Based on the above findings, the therapeutic efficacy of CBP‐Exos in CCL_4_‐induced ALI in mice has been elucidated, along with its potential mechanisms. Moving forward, we sought to determine whether CBP‐Exos exert therapeutic effects in chronic liver injury models, thereby expanding their potential applicability. Parenchymal liver fibrosis was induced through biweekly intraperitoneal injections of CCl_4_ over a 6‐week period. Therapeutic intervention involved administration of CBP‐Exos starting 3 weeks postinitial CCl_4_ injection, followed by biweekly doses (Figure 7A). CBP‐Exos treatment visibly reversed the rough texture of the liver observed in the CCl_4_ group (Figure 7B). Obviously, H&E staining revealed prominent hepatic fiber bands in the CCl_4_ group, while CBP‐Exos treatment significantly inhibited fiber band formation and reduced inflammatory cell infiltration (Figure 7C). Sirius Red staining and RT‐qPCR analysis were employed to assess liver fibrosis in treated mice. Compared with the CCl_4_ group, CBP‐Exos treatment demonstrated significant antifibrotic effects, evidenced by reduced Sirius Red‐positive areas and markedly reduced Col1a1 expression (Figures 7D and S16A). Additionally, CCl_4_‐treated mice displayed increased hepatic expression of proinflammatory factors, including TNF‐α, IL‐1β, and IL‐6, while CBP‐Exos treatment significantly reduced the levels of these inflammation‐related proteins in liver tissues (Figure 7E).

Given the significant role of the hepatic immune microenvironment in fibrosis development [43], immune cell populations were analyzed by flow cytometry. This investigated the impact of CBP‐Exos treatment on the liver immune microenvironment. Flow cytometry analysis revealed a significant reduction in F4/80+ macrophage populations following CBP‐Exos treatment compared with the CCl_4_ group (Figure 7F,G). Proportions of other intrahepatic CD45+ immune cells, such as CD4+/CD8+ T lymphocytes, neutrophils, NK cells, and DCs, were largely unaltered (Figure S16B–E). To ascertain whether CBP‐Exos directly influence macrophage subsets, flow cytometry analysis of M1 (CD80+) and M2 (CD206+) polarization was performed. This analysis revealed that CBP‐Exos treatment significantly increased the ratio of anti‐inflammatory M2‐type to proinflammatory M1‐type macrophages (Figure 7H–J). Above all, our results indicate that CBP‐Exos treatment could inhibit the progression of liver fibrosis by reducing collagen deposition, reducing systemic inflammatory effects, and improving the liver immune microenvironment in CCl_4_‐induced chronic liver injury.

## 3 Discussion

Liver injury, induced by various factors such as viral hepatitis, drug abuse, and excessive alcohol consumption, can lead to acute or chronic liver damage, ultimately resulting in severe conditions like liver failure, cirrhosis, and hepatocellular carcinoma, which pose substantial risks to human health and safety [44, 45]. Liver transplantation currently remains the most effective treatment for advanced liver damage, but challenges such as a shortage of organ donors and the lifelong need for immunosuppressive therapy persist [46]. In recent years, exosomes have emerged as a promising alternative in the treatment of liver injury [47, 48]. Most studies have focused on exosomes derived from MSCs, with some studies investigating exosomes from human liver stem cells [49, 50, 51], hepatocytes [52, 53], amniotic epithelial cells [54], and even from normal mouse plasma for liver injury treatment [26]. Due to the varying effects of plasma‐derived exosomes from different age groups, we conducted a comparative study on the hepatoprotective effects of CBP‐Exos, YBP‐Exos, and OBP‐Exos in ALI mice model. Our findings revealed that, compared with YBP‐Exos, CBP‐Exos exhibited superior capabilities in inhibiting liver necrosis and oxidative stress, while OBP‐Exos showed no therapeutic benefit and may even exacerbate liver injury. Additionally, our further study confirmed the significant antioxidative and antiapoptotic effects of CBP‐Exos in liver injury, identifying the molecular mechanisms through which CBP‐Exos alleviate hepatocyte oxidative damage, notably by transferring miR‐410‐3p. Consistent with this, direct miR‐410‐3p agomir treatment significantly inhibited the pathological progression of ALI. Moreover, chronic liver injury results further validated the enduring efficacy of CBP‐Exos, demonstrating their ability to inhibit fibrosis progression and regulate intrahepatic immune responses. The pathological continuum between acute and chronic liver injury is largely mediated by mitochondrial dysfunction, immune cell activation, and dysregulated repair mechanisms [55, 56]. The chronic liver injury findings illustrate that interventions targeting acute damage mechanisms, such as oxidative stress and apoptosis, can also effectively modulate chronic injury processes like fibrosis and immune activation. Consequently, this dual‐model approach substantiates the robustness of the CBP‐Exos‐mediated protective mechanisms and highlights their clinical relevance for liver disease treatment across different pathological stages.

Numerous studies have demonstrated that the liver is the primary organ for the uptake of EVs after intravenous administration. This process relies on the internalization of bioactive cargo. Such cargo, including miRNAs and proteins, plays a crucial role in regulating liver injury repair [51, 57]. In this study, intravenous administration of DiR‐labeled CBP‐Exos into mice revealed their predominant enrichment within the liver. Accumulation was significantly enhanced in livers afflicted by CCl_4_‐induced injury. This preferential uptake in damaged tissue was supported by in vitro coculture experiments using PKH26‐labeled CBP‐Exos and hepatocytes. These findings affirm that CBP‐Exos are preferentially recruited to the liver following liver injury, which is a key reason for their suitability in the treatment of liver‐related diseases.

Exosomes are known to contain proteins, lncRNAs, and miRNAs, which are transported through systemic circulation. Several proteins and lncRNAs encapsulated in exosomes have been identified for their hepatoprotective effects, such as lncRNA19, chaperonins CCT2, and glutathione peroxidase GPX1 [58, 59, 60]. In recent years, exosomal miRNAs have garnered increasing attention due to their potential for producing substantial effects even in minute quantities. Growing evidence has highlighted the hepatoprotective roles of exosomal miRNAs derived from stem cells [61, 62]. In this study, high‐throughput sequencing was utilized to compare the miRNA expression profiles of CBP‐Exos and OBP‐Exos. Subsequent RT‐qPCR analysis led to the identification of six miRNAs exhibiting differential expression. Using an in vitro model of hepatocyte oxidative injury, corresponding miRNA mimics and inhibitors were synthesized and employed. Among the six differentially expressed miRNAs upregulated in CBP‐Exos, miR‐410‐3p was found to exhibit significant hepatoprotective effects in vitro. Notably, treatment with miR‐410‐3p agomir also demonstrated remarkable improvements in hepatic function and hepatoprotective capacity in vivo experiments. These results collectively suggest that the hepatoprotective effects of CBP‐Exos are primarily attributed to miR‐410‐3p.

Mitochondria‐mediated apoptosis has been identified as a crucial mechanism in hepatocyte oxidative damage. Oxidative stress triggered by agents such as APAP, CCl_4_, and LPS can lead to alterations in mitochondrial permeability, mitochondrial dysfunction, and an increase in cell apoptosis, ultimately resulting in ALI and hepatotoxicity [63]. Exosomes derived from MSCs have shown hepatoprotective properties, particularly in restoring mitochondrial function by delivering mitochondria and antioxidant proteins [64, 65, 66]. Recent studies have indicated that oxidative damage and mitochondrial dysfunction in hepatocytes caused by APAP can be mitigated through the Nrf2‐mediated JNK/CytoC/caspase‐3 signaling pathway following treatment with Honeysuckle Lonicera and Dendrobium bioalkaloids [67, 68]. Additionally, it has been demonstrated that PUMA, a member of the Bim protein family, is significantly upregulated through the RIP1/JNK‐dependent pathway, contributing to mitochondrial dysfunction and the release of mitochondrial cell death factors in APAP‐induced liver injury [69]. However, the molecular mechanism by which miR‐410‐3p targets Bim in liver injury remains unexplored. In our study, we observed that miR‐410‐3p directly targets Bim for posttranscriptional degradation, leading to significant upregulation of the antiapoptotic protein Bcl2 and decreased expression and release of mitochondrial CytoC. Additionally, we observed decreased MPTP opening and reduced leakage of proinflammatory mtDNA. These effects collectively contributed to the alleviation of oxidative damage in hepatocytes. Interestingly, a recent study showed that small EVs derived from young plasma could reverse age‐related dysfunction by enhancing mitochondrial energy metabolism [25]. Similarly, another study demonstrated that the inhibition of mitochondria‐mediated apoptosis improved healthspan in aged mice by reducing the release of mtDNA [70]. This suggests a potential link between aging, mitochondria, and apoptosis. Our findings further support that CBP‐Exos alleviate liver injury by modulating mitochondria‐mediated apoptosis signaling. Nevertheless, whether CBP‐Exos can be involved in regulating cellular senescence to mediate liver repair needs further study.

In recent years, numerous preclinical studies have shown promising potential for miRNAs in treating various major diseases, with several miRNA‐targeted drugs advancing to the preclinical stage. Examples include miRNA‐21 inhibitors for chronic kidney disease, miRNA‐29 for treating keloids, miRNA‐155 inhibitors for cutaneous T‐cell lymphoma, miRNA‐17 for polycystic kidney disease, miRNA‐34a for various cancers, and miRNAs like miR‐15, miR‐195, miR‐29, and miR‐378 for cardiac diseases [71, 72, 73]. Despite this progress, clinical trials of miRNA‐based drugs for liver disease have not yet been explored.

Currently, the clinical applications of UCB mainly focus on hematopoietic stem cell transplantation (HSCT). Since the establishment of the UCB Bank in the United States in 1991 and its opening in France in 1995, over 805,000 cryopreserved UCB units have been made available for use, as retrieved from the WMDA database [74, 75, 76]. Nowadays, both public and private cord blood banking businesses are developing rapidly and UCB is mainly used for HSCT in clinical practice, while the development and utilization of umbilical CBP remain limited [77, 78]. It has been reported that during the processing of UCB, nonhematopoietic cells and plasma components can be relatively easily separated [79, 80, 81]. In addition to enhancing the clinical application of cellular resources in UCB, exploring the clinical value of noncellular components, especially plasma, is of great importance. Numerous studies have demonstrated that CBP holds potential for clinical applications, particularly in antiaging therapies [8, 19, 20, 82]. Furthermore, several studies have highlighted the potential clinical value of CBP‐Exos in tissue repair and wound healing [83, 84]. Our study indicates that CBP‐Exos may have promising clinical potential in the treatment of liver diseases.

While our study provides significant insights into the hepatoprotective effects of CBP‐Exos and mir‐410‐3p, there are notable limitations that warrant discussion. First, the reliance on animal models restricts the translational relevance of our findings to human liver diseases. Due to the difficulty in obtaining clinical samples of human hepatocytes and the challenges associated with expanding PHs in vitro, the therapeutic efficacy of CBP‐Exos has not yet been validated in human hepatocyte models. Moving forward, we plan to utilize induced pluripotent stem cell technology to acquire human liver organoid model to further assess the clinical potential of CBP‐Exos in treating severe liver injury. This will help us further validate the clinical translation potential of CBP‐Exos in the treatment of severe liver injury. Another notable limitation of this study is the lack of direct comparison between the miRNA expression profiles of CBP‐Exos and YBP‐Exos. While both exosome types demonstrated similar therapeutic effects on liver injury, the molecular basis for their shared capabilities remains unclear. Future investigations should explore whether certain miRNAs exhibit overlapping patterns between CBP‐Exos and YBP‐Exos, and whether developmental stage or age‐related factor contribute to any observed differences. Such analyses would provide valuable insights into the mechanisms driving the therapeutic potential of plasma‐derived exosomes from different sources. Notably, while this study primarily focused on parenchymal hepatic cells, we speculate that Kupffer cells might also indirectly contribute to the therapeutic effects of CBP‐Exos. Kupffer cells are not only initiators of inflammation but also play an important role in mitigating inflammatory damage and tissue repair. For instance, in models like ischemia/reperfusion injury, Kupffer cells have been shown to exert hepatoprotective effects by promoting the activation of M2 macrophages [85, 86]. In addition, during liver injury, there is a close mutual regulation between parenchymal hepatic cells and Kupffer cells, and this communication is largely mediated by intercellular EVs [87]. Damaged hepatic parenchymal cells release damage‐associated molecules that activate Kupffer cells, while activated Kupffer cells produce proinflammatory cytokines that, in turn, exacerbate hepatocyte damage, creating a vicious cycle. Conversely, during inflammation resolution and repair, intercellular signaling may promote tissue recovery. The observed therapeutic effect of CBP‐Exos in the CCl_4_‐induced chronic liver injury model in this study, characterized by reduced fibrosis and inflammation, might be partly attributable to CBP‐Exos modulating this complex interplay between hepatocytes and Kupffer cells. Therefore, future studies can further explore the direct effects of CBP‐Exos on Kupffer cells and their contribution to the overall therapeutic mechanism. Addressing these limitations is crucial for advancing the application of CBP‐Exos in liver disease therapy.

## 4 Conclusion

In conclusion, CBP‐Exos exhibited superior therapeutic effects in models of both ALI and chronic liver injury induced by CCl_4_. Mechanistically, miR‐410‐3p, identified as a primary hepatoprotective component of CBP‐Exos, targets the proapoptotic gene Bim for posttranscriptional degradation. This process upregulates the antiapoptotic protein Bcl2 and reduces the expression and release of mitochondrial CytoC. Furthermore, it decreased the leakage of proinflammatory mtDNA and restored mitochondrial structure and function. These effects collectively inhibited oxidative stress and hepatocyte apoptosis caused by oxidative damage. Significantly, in vivo data demonstrated that overexpression of miR‐410‐3p effectively ameliorated CCl_4_‐induced liver injury. Our findings present a novel mechanism underlying CBP‐Exos treatment for liver injury with exosomal miR‐410‐3p as a novel hepatoprotection molecular and highlight the therapeutic potential of miR‐410‐3p in the prevention and therapy of liver disease.

## 5 Materials and Methods

### 5.1 Sample Collection and Exosome Isolation

UCB, young blood, and old blood samples were collected from healthy donors. Plasma was separated and subjected to sequential centrifugation and ultracentrifugation to isolate exosomes. Exosome pellets were washed, filtered (0.22 µm), and resuspended in PBS for storage at −80°C or immediate use. Exosome characterization was performed via nanoparticle tracking analysis, TEM, and Western blot for specific markers (TSG101, CD63, Alix).

### 5.2 Cell Culture and Treatments

Mouse AML12 hepatocytes (ATCC) and PHs (isolated from C57BL/6 mice as previously described) were cultured in appropriate media. For in vitro assays, cells were incubated with CBP‐Exos (10 µg/mL) and/or subjected to H_2_O_2_‐induced oxidative stress. Exosome uptake was assessed using PKH26 or DiR‐labeled exosomes and confocal microscopy.

### 5.3 Animal Models

Female C57BL/6 mice were used for in vivo studies. ALI was induced by intraperitoneal injection of CCl_4_ (0.5 µL/g body weight). CBP‐Exos (10 µg/g) were administered intravenously at 0 and 1 days postmodeling. For liver fibrosis, CCl_4_ was administered twice weekly for 6 weeks, with CBP‐Exos administered during the last 3 weeks.

### 5.4 Molecular and Cellular Analyses

Exosomal and cellular RNA was extracted for high‐throughput miRNA sequencing or RT‐qPCR. miRNA differential expression was analyzed as described in the Supporting Information. Apoptosis and ROS assays (TUNEL, Annexin V/7‐AAD, DCFH‐DA), mitochondrial function (mitotracker, TEM), and immunofluorescence staining were performed following standard protocols. Dual‐luciferase reporter assays were used to confirm miRNA target genes.

### 5.5 Statistical Analysis

All statistical analyses were performed using GraphPad Prism 8.0. Statistical significance was analyzed using the one‐way AVONA. Differences were considered statistically significant at *p* < 0.05. The symbols used to denote significance are as follows: *p < 0.05, **p < 0.01, ***p < 0.001, ****p < 0.0001, and ns (no statistical significance). Details of antibodies, primer sequences, and additional methods are provided in the Supporting Information.

## Author Contributions

Lin Zhang designed the study, performed the experiments, analyzed the data, and wrote the manuscript. Yushuang Ren carried out the experiments and analyzed the data. Dongsheng Su helped with in vivo experiments; Qingyuan Jiang provided human UCB. Huan Peng helped with in vitro experiments. Fuyi Cheng, Hantao Zhang, Xue Bai, Xiao Wei, Weixiao Yang, Pusong Zhao, and Yixin Ye helped to complete experiments including flow cytometry and IHC assay. Gang Shi helped to interpret data and revised the manuscript. Hongxin Deng was involved in obtaining funding and study supervision. All authors have read and approved the final manuscript.

## Ethics Statement

All animal care and procedures are in accordance with the standards of “Regulations of Sichuan Province on the Feeding and Management of Medical Experimental Animals” and “Regulations of Sichuan University on the Management of Experimental Animals.” All the authors were compliant with all relevant ethical regulations. All animal experiments were approved by the Medical Ethics Committee of Sichuan University (No. 2020401A). The human blood samples were collected from Sichuan Provincial Hospital for Children and Women. According to the approval of the Medical Ethics Committee of West China Hospital, Sichuan University (2021‐167), human UCB samples (50–60 mL per sample) were obtained from umbilical veins after healthy neonatal delivery with permission from the infants’ parents and Sichuan Provincial Hospital for Children and Women. The whole blood was collected in a multiple‐system bag containing citrate phosphate dextrose as an anticoagulant. All the authors compliance with all relevant ethical regulations.

## Conflicts of Interest

The authors declare no conflicts of interest.

## Supporting information




**Supporting File**: mco270339‐sup‐0001‐SuppMat.pdf

## Data Availability

The miRNA high‐throughput sequencing data accessible at NCBI SRA database, accession SRP447247. All data are included in this published article and its Additional information file.
