# Supplementary material for: Human Umbilical Cord Blood Plasma‐Derived Exosomal miR‐410‐3p Alleviates Liver Injury by Regulating the Mitochondria‐Mediated Antiapoptotic Signaling
Source: MedComm (2020). 2025 Aug 24;6(9):e70339. doi: 10.1002/mco2.70339 (PMC12375691; doi:10.1002/mco2.70339)
Supplement: Supplementary file 1 — Supporting File: mco270339‐sup‐0001‐SuppMat.pdf [file MCO2-6-e70339-s001.pdf]

Human umbilical cord blood plasma-derived exosomal miR-410-3p alleviates liver injury by regulating the mitochondria-mediated anti-apoptotic signaling

Lin Zhang [1]#, Yushuang Ren [1]#, Dongsheng Su[1]#, Qingyuan Jiang[2], Huan Peng[3], Fuyi Cheng[1], Hantao Zhang[4], Xue Bai[1], Xiao Wei[1], Weixiao Yang[1], Pusong Zhao[1], Yixin Ye[1], Gang Shi[1], Hongxin Deng[1]*

1 Department of Biotherapy, Cancer Center and State Key Laboratory of Biotherapy, West China Hospital, Sichuan University, Chengdu, China.

2 Department of Obstetrics, Sichuan Provincial Hospital for Women and Children, Chengdu, China.

3 Department of Clinical Laboratory, Sichuan Provincial Hospital for Women and Children, Chengdu, China.

4 West China Biobank, West China Hospital, Sichuan University, Chengdu, China.

†These authors contributed equally to this work.

Conflicts of interest: There are no conflicts of interest to disclose by any of the authors.

* Correspondence to: Hongxin Deng, denghongx@scu.edu.cn, Department of Biotherapy, Cancer Center and State Key Laboratory of Biotherapy, West China Hospital, Sichuan University, Chengdu, Sichuan, 610041, the People's Republic of China.

Table S1. List of primers used in this study.

Gene	Forward primer (5' to 3')	Reverse primer (5' to 3'
Mouse-CCN2	CACTCTGCCAGTGGAGTTCA	AAGATGTCATTGTCCCCAGG
Mouse-Cola1	GCCCGAACCCCAAGGAAAAG AAGC	CTGGGAGGCCTCGGTGGACATT AG
Mouse-α-SMA	GGCTCTGGGCTCTGTAAGG	CTCTTGCTCTGGGCTTCATC
Mouse-GAPDH	TGCACCACCAACTGCTTAGC	GGCATGGACTGTGGTCATGAG
Mouse-ALB	CAGCGGAGCAACTGAAGACT	GGTTTGGACCCTCAGTCGAG

Supplementary Figures:

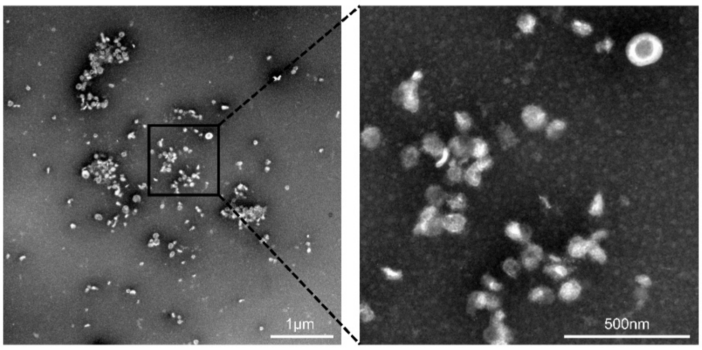

Figure S1. Morphology and size identification of CBP-Exos by TEM.

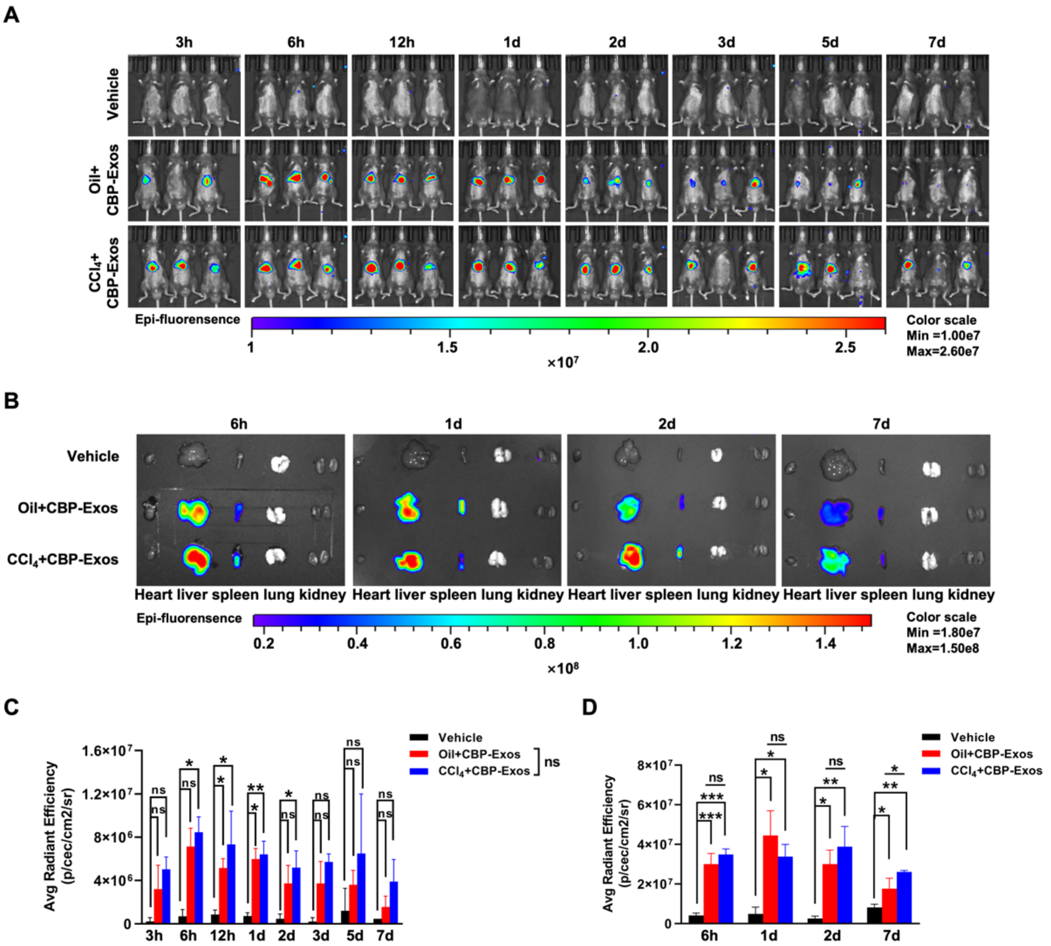

Figure S2. Distribution of CBP-Exos in mice after tail intravenously injection.

(A) Representative IVIS images and (C) quantitive analysis of 3 h, 6 h, 12 h, 1 d, 2 d, 3 d, 5d, and 7d post-injection of DiR-labeled CBP-Exos. (B) Representative organ distribution images and (D) quantitative analysis of 3 h, 6 h, 12 h, 1 d, 2 d, 3 d, 5 d, and 7 d post-injection of DiR-labeled CBP-Exos. Data are presented as mean ± SD. One-

way AVONA: ns, not significance; *p < 0.05, **p < 0.01 and ***p < 0.001.

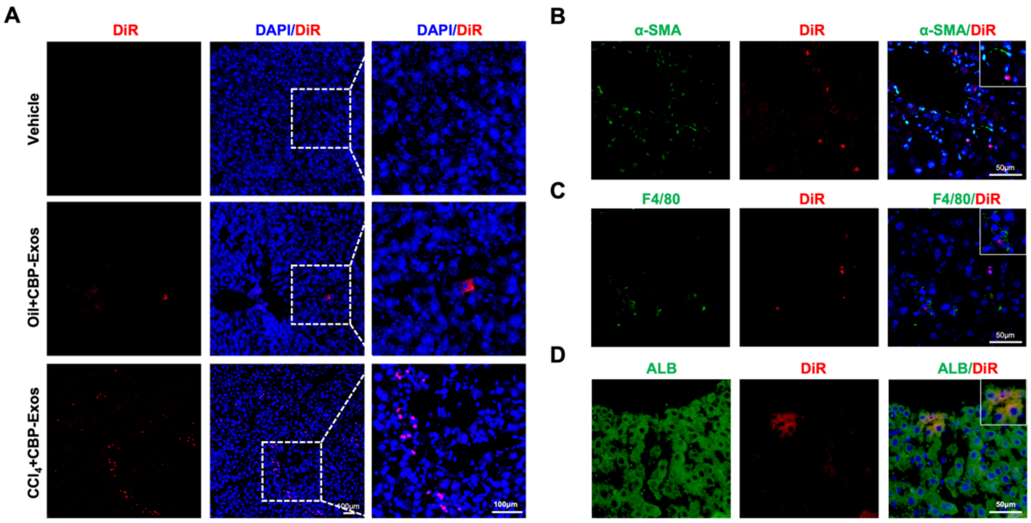

Figure S3. Distribution and absorption of CBP-Exos in mouse liver. (A) Representative immunofluorescence staining images of the distribution and location of DiR-labeled CBP-Exos in mouse liver. (B) Representative immunofluorescence staining images of DiR-labeled CBP-Exos and hepatic stellate cells (α-SMA) in mouse liver. (C) Representative immunofluorescence staining images of DiR-labeled CBP-Exos and macrophages (F4/80) in mouse liver. (C) Representative immunofluorescence staining images of DiR-labeled CBP-Exos and hepatocytes (ALB) in mouse liver.

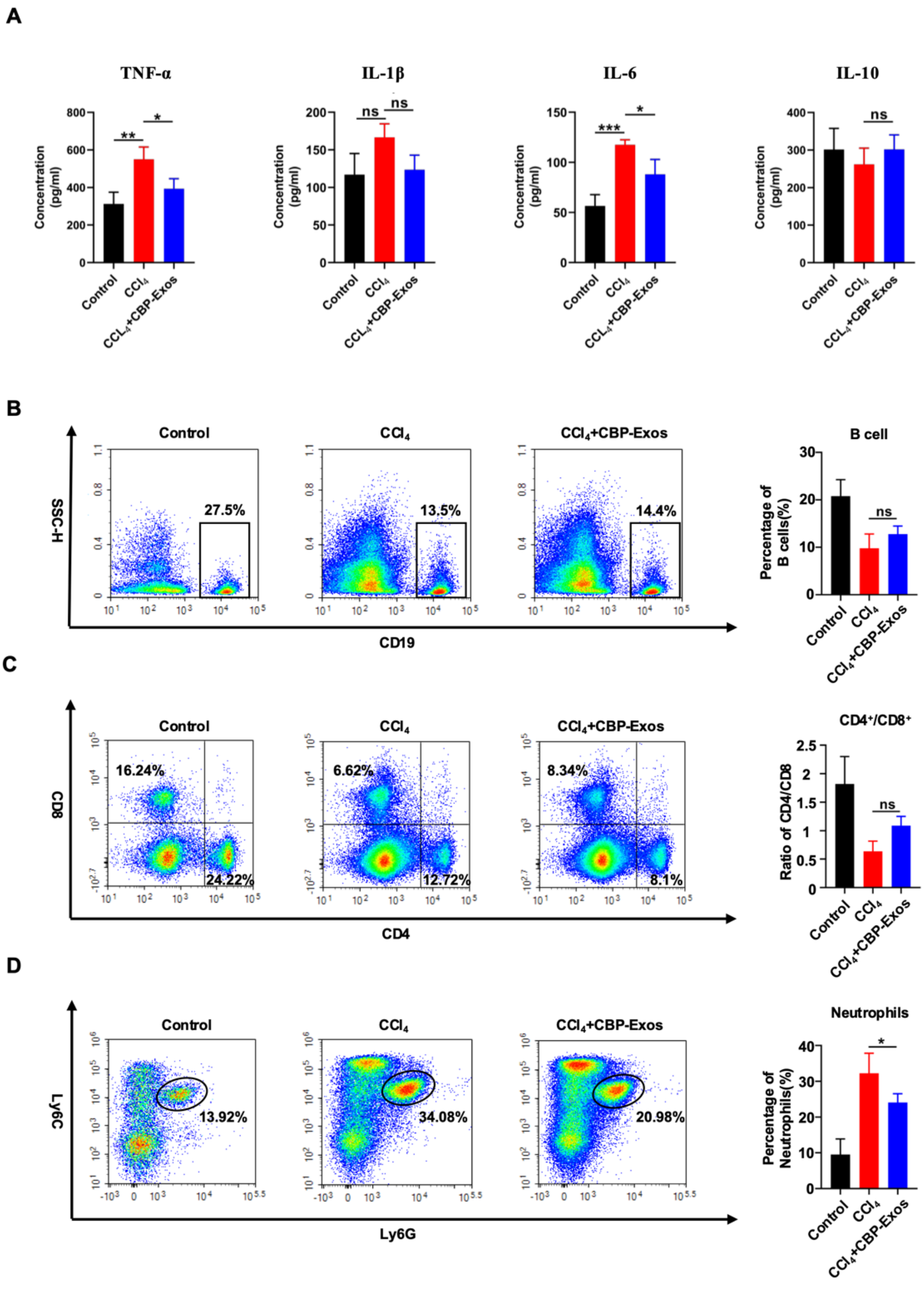

Figure S4. Inflammatory regulation of CBP-Exos in ALI mice. (A)Serum inflammatory cytokines (TNF-α, IL1-β, IL-6, and IL-10) were determined by Elisa Detection Kit (Control group and CCl_4 group, n=3; CCl_4+CBP-Exos group, n=4). (B) Flow cytometry analyses and quantitative analysis of B cells in intrahepatic CD45+ immune cells of each treatment group (n=5). (C) Flow cytometry analyses and quantitative analysis of the ratio of CD4+/CD8+ cells in intrahepatic CD45+ immune cells of each treatment group (n=5). (D) Flow cytometry analyses and quantitative analysis of neutrophil cells in intrahepatic CD45+ immune cells of each treatment group

(n=5). Data are presented as mean ± SD. One-way AVONA: ns, not significance; *$p < 0.05$, **$p < 0.01$ and ***$p < 0.001$.

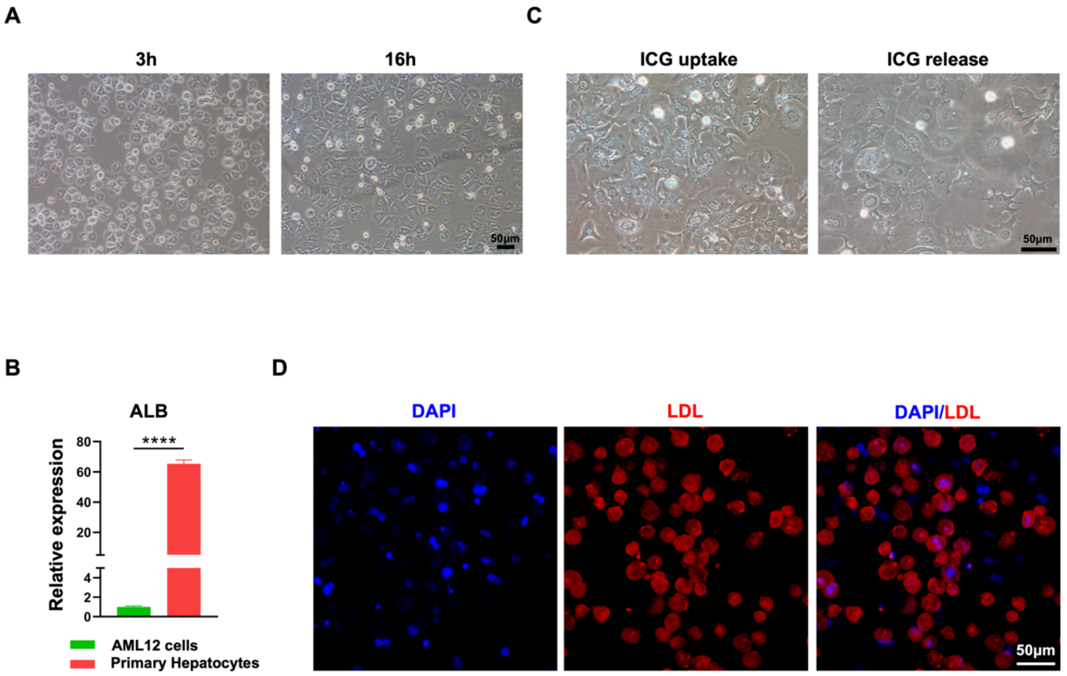

Figure S5. Identification of isolated primary hepatocytes (PHs). (A) The cell morphology of PHs at 3 h and 16 h after adhering to the wall. (B) RT-qPCR analysis of the expression of ALB in PHs compared to AML12 hepatocytes (n=3). (C) ICG uptake and release assay was used to detect the reserve function of hepatocytes. (D) Immunofluorescence images of LDL up-taken were used to detect the metabolic function of hepatocytes.

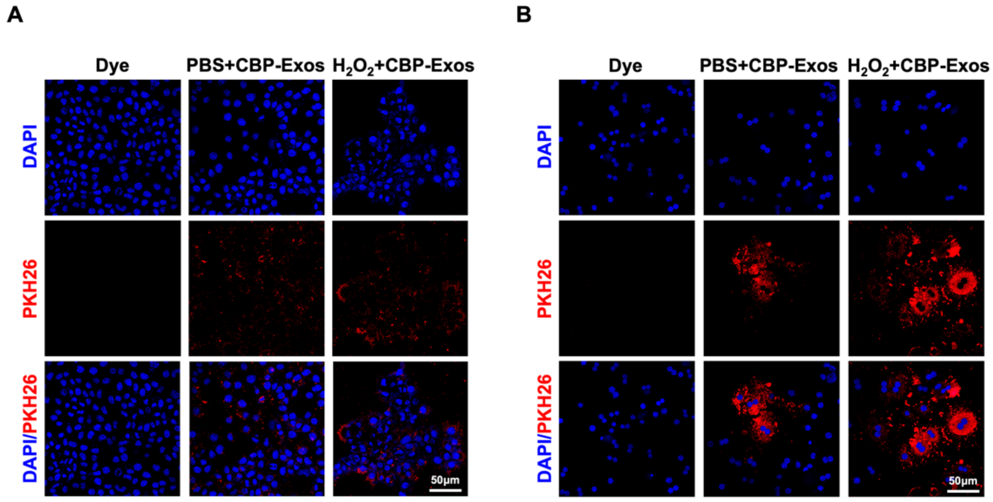

Figure S6. Uptake of CBP-Exos in hepatocytes in vitro. (A) Immunofluorescence images of the PKH26-labeled CBP-Exos up taken by AML12 hepatocytes. (A) Immunofluorescence images of the PKH26-labeled CBP-Exos up taken by PHs.

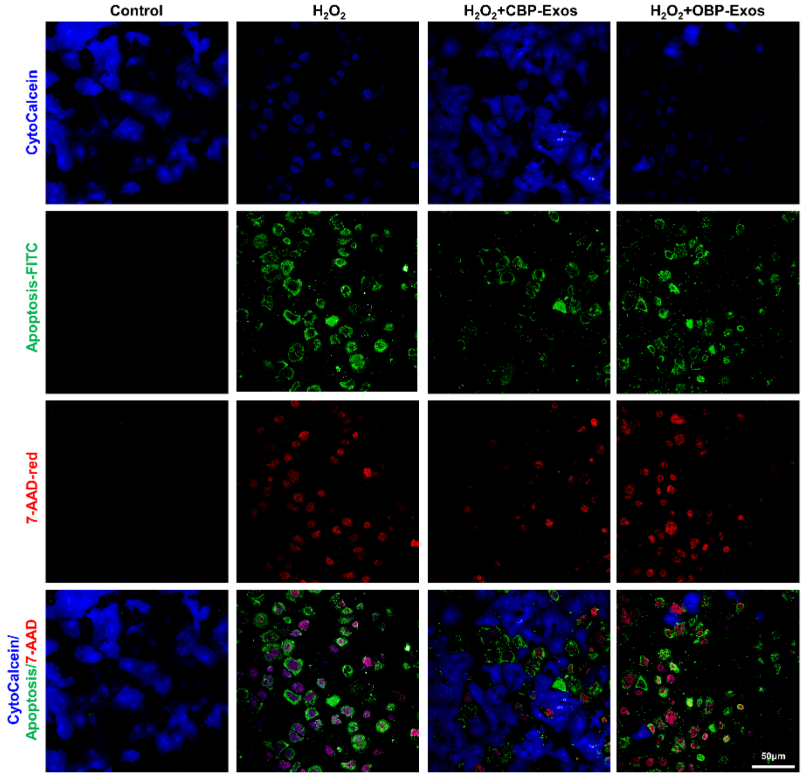

Figure S7. Immunofluorescence analysis of live cells (Cytocalcein-Blue), early apoptotic cells (Annexin V-FITC), and early apoptotic cells or necrosis(7-AAD-Red) in PHs treated with CBP-Exos or OBP-Exos, respectively.

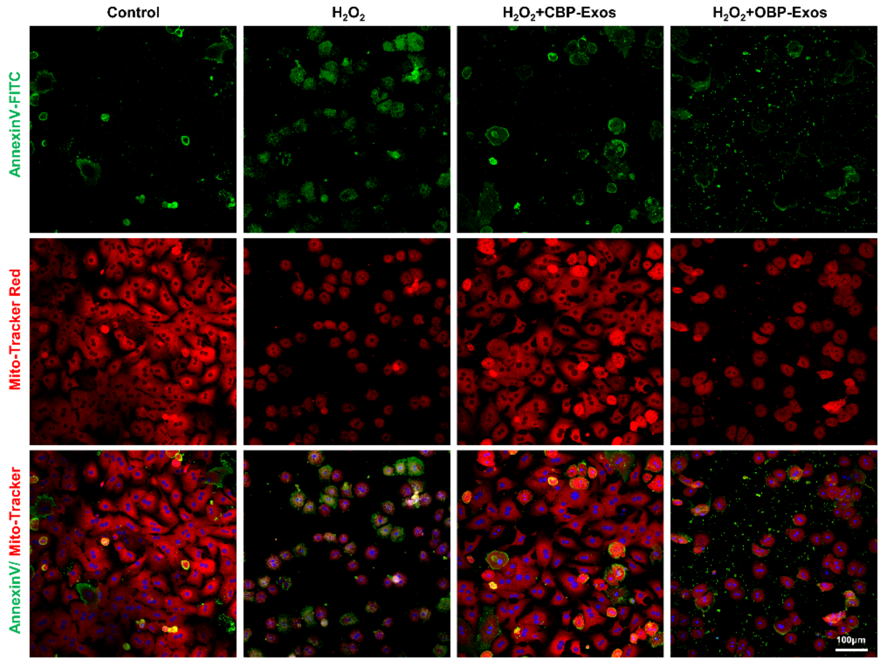

Figure S8. Mitochondrial membrane potential and apoptosis staining images of AML12 hepatocytes treated with CBP-Exos or OBP-Exos, respectively.

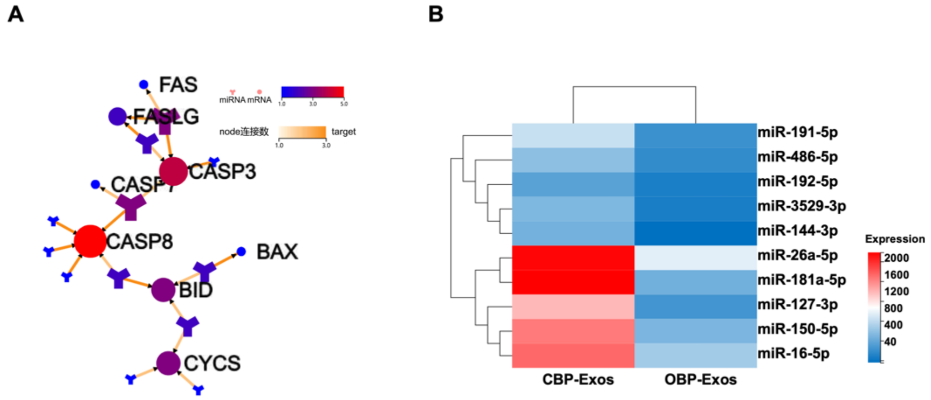

Figure S9. MiRNAs profiling assays analysis of CBP-Exos and OBP-Exos. (A) MiRNA-mRNA regulatory network of differential miRNAs in CBP-Exos and OBP-Exos and their target genes involved in liver injury apoptosis signaling pathway(n=3). (A) Heat maps of the top ten differential miRNAs in CBP-Exos (fold change>2.0，n=3).

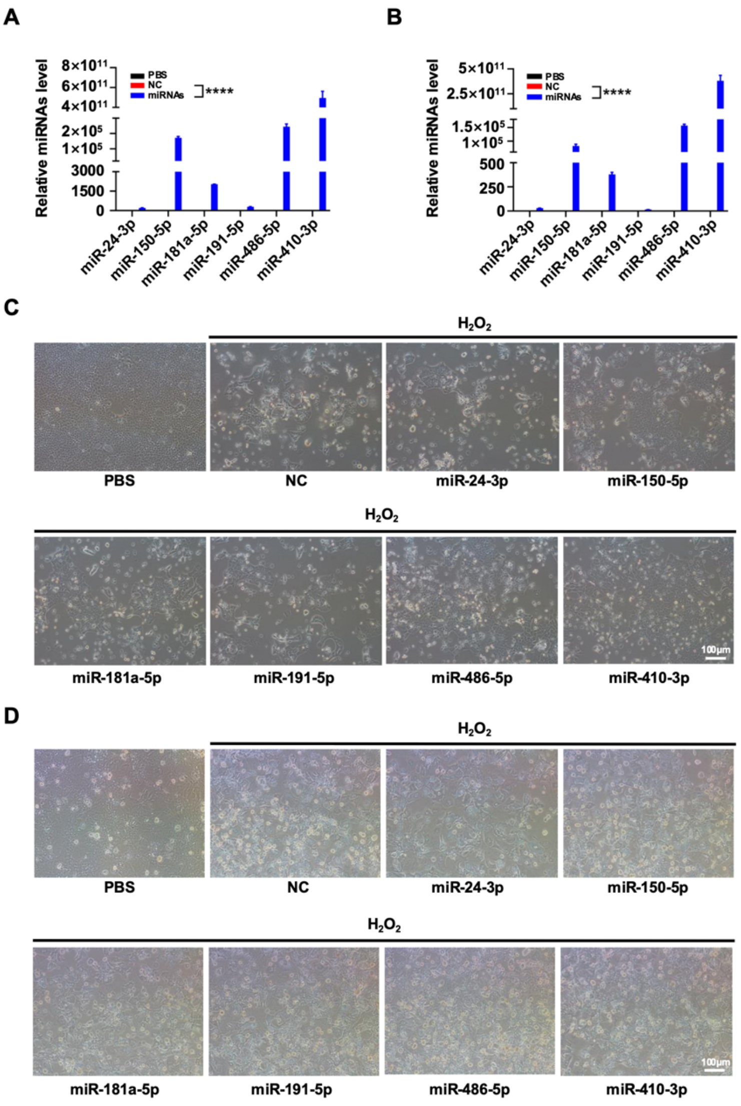

Figure S10. miRNAs mimic transfection in hepatocytes. RT-qPCR analysis of the 6 differentially expressed miRNAs in AML12 hepatocytes(A) and PHs(B) after transfecting with the corresponding miRNAs. The cell morphology changes of AML12 hepatocytes(C) and PHs(D) induced by H_2O_2 after transfecting with the corresponding miRNAs.

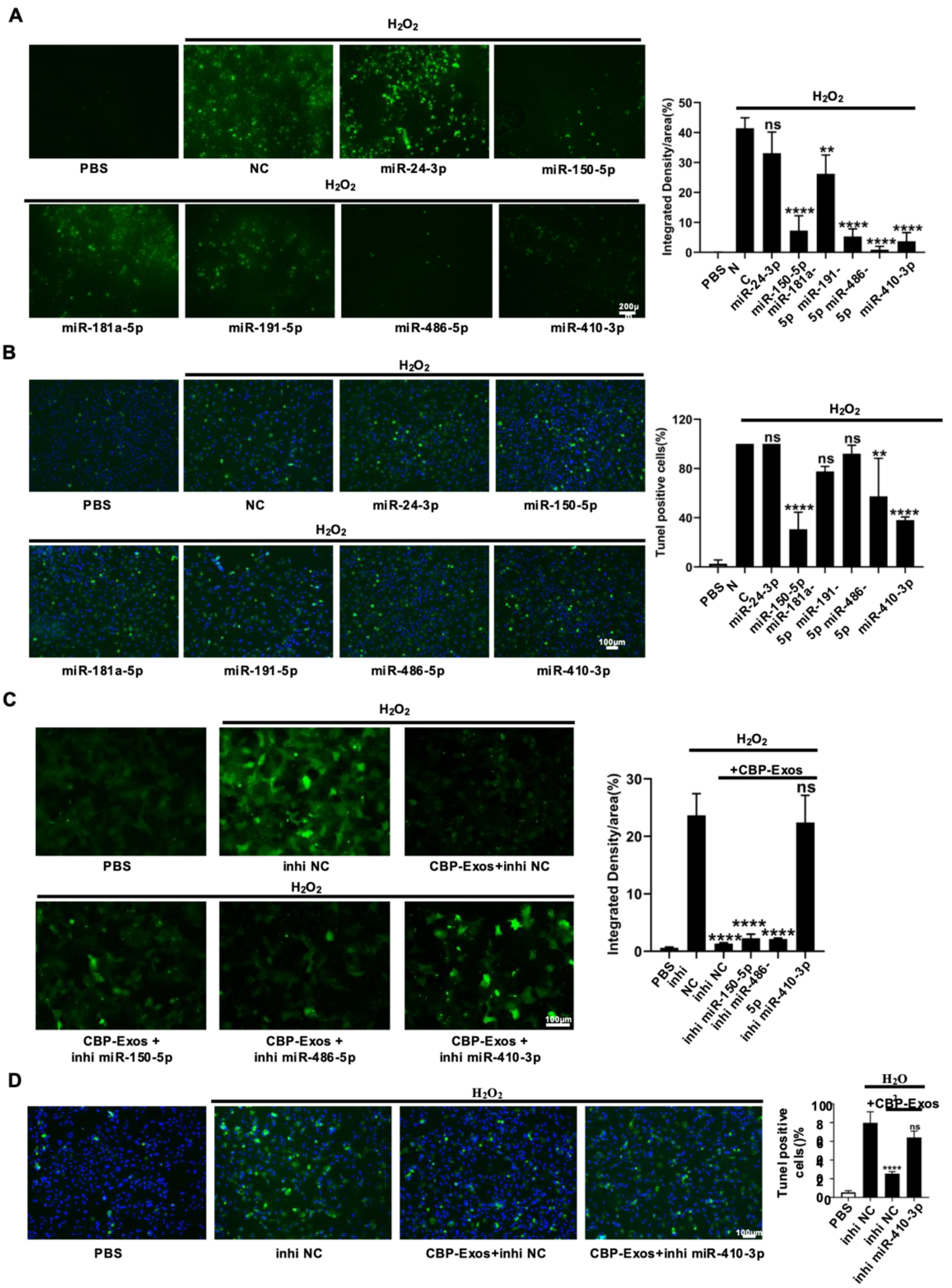

Figure S11. The anti-oxidative and anti-apoptotic function of miR-410-3p on H_2O_2-stimulated PHs. (A) Immunofluorescence analysis of ROS-positive cells in PHs cells transfected with a mimic of indicated miRNA or negative control (NC) for 24 hours followed by H_2O_2-stimulated hepatocytes (n=3). (B) The TUNEL staining analysis of apoptosis of PHs transfected with a mimic of indicated miRNA or negative control (NC) for 24 hours followed by H_2O_2-stimulated hepatocytes (n=3). (C) Immunofluorescence analysis of ROS-positive cells in PHs preincubated with CBP-Exos and transfected with an inhibitor of indicated miRNA or negative control (inhiNC)

for 24 hours followed by H_2O_2-stimulated hepatocytes (n=3). (D) The TUNEL staining analysis of apoptosis of PHs preincubated with CBP-Exos and transfected with miRNA-410-3p inhibitor or negative control (inhiNC) for 24 hours followed by H_2O_2-stimulated hepatocytes (n=3). Data are presented as mean ± SD. One-way AVONA: ns, not significance; $**p < 0.01$ and $****p < 0.0001$.

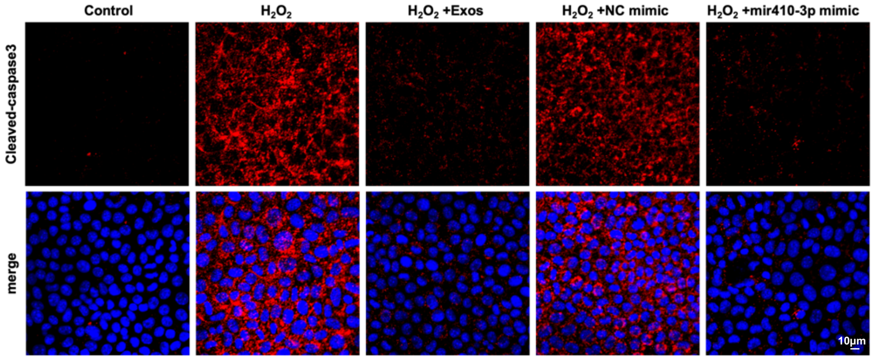

Figure S12: Representative immunofluorescence images of the cleaved-caspase3 in H_2O_2-stimulated AML12 hepatocytes treated with CBP-Exos, negative control (NC), miR-410-3p mimic or mimic negative control (NC mimic).

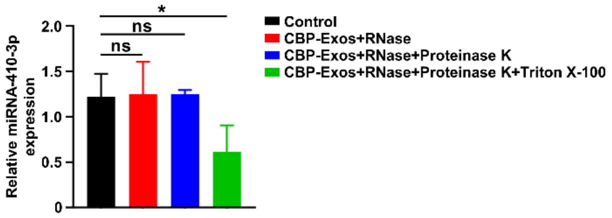

Figure S13. RT-qPCR analysis of miR410-3p expression in CBP-Exos incubated with PBS (control), RNase, RNase+Proteinase K, or RNase+ProteinaseK+Triton X-100 for 45 minutes at 37° C. RNase (10 μg/ml); Proteinase K (100 μg/ml); Triton X-100 (0.3%). Data are normalized to spiked-in cel-miR-39 (n=3). Data are presented as mean ± SD. One-way AVONA: ns, not significance; $*p < 0.05$.

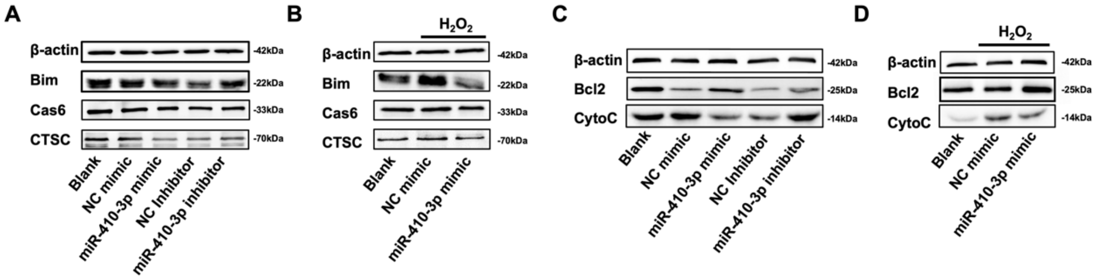

Figure S14. The targets and downstream signals of miR-410-3p on PHs. (A) Western blots analysis showing protein expressions of the three putative miR-410-5p target genes in AML12 hepatocytes treated with miR-410-3p mimic, mimic negative control (NC mimic), miR-410-3p inhibitor, or inhibitor NC. (B) Western blots analysis showing protein expressions of the three putative miR-410-5p target genes in H_2O_2-stimulated AML12 hepatocytes treated with miR-410-3p mimic or mimic negative control (NC mimic). (C) Western blots analysis showing protein expressions of Bcl2 and CytoC in AML12 hepatocytes treated with miR-410-3p mimic, mimic negative control (NC mimic), miR-410-3p inhibitor, or inhibitor NC. (D) Western blots analysis showing protein expressions of Bcl2 and CytoC in H_2O_2-stimulated AML12 hepatocytes treated with miR-410-3p mimic or mimic negative control (NC mimic).

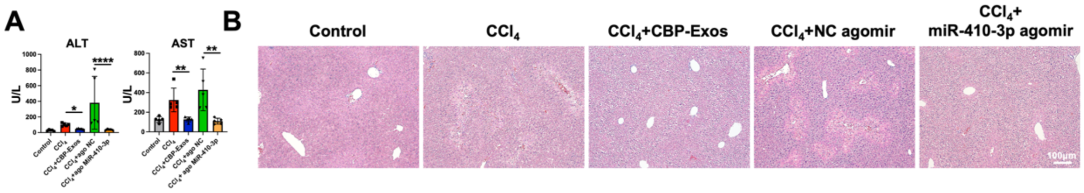

Figure S15: miR-410-3p agomir alleviates APAP-induced acute liver injury. (A) Serum levels of Alanine aminotransferase (ALT) and Aspartate aminotransferase (AST) after 1 d in different treatment groups (n=5); (B) Representative images of Hematoxylin and eosin (H&E) staining of liver sections.

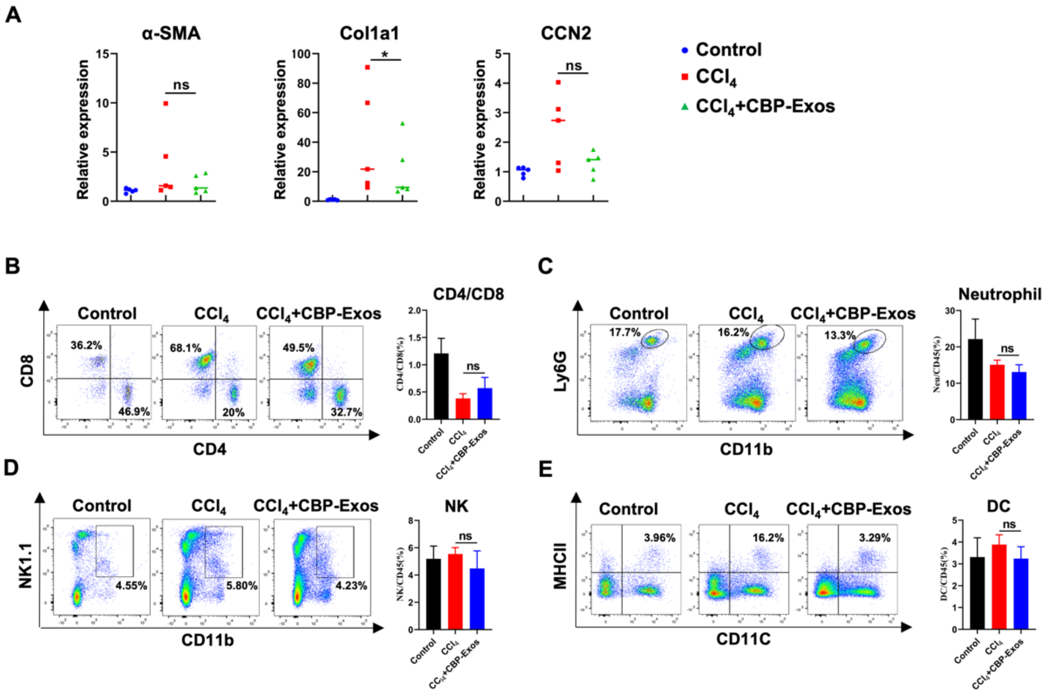

Figure S16. Inflammatory regulation of CBP-Exos in LF mice. (A) The mRNA expression levels of liver fibrosis-related genes (Alpha smooth muscle actin, α-SMA; Collagen type I alpha 1, Col1a1 and Connective tissue growth factor 2, CCN2) in liver tissues (n=5). (A) Flow cytometry analyses and quantitative analysis of the ratio of CD4+/CD8+ cells in intrahepatic CD45+ immune cells of each treatment group (n=6). (C) Flow cytometry analyses and quantitative analysis of neutrophil cells in intrahepatic CD45+ immune cells of each treatment group (n=6). (D) Flow cytometry analyses and quantitative analysis of NK cells in intrahepatic CD45+ immune cells of each treatment group (n=6). (E) Flow cytometry analyses and quantitative analysis of DC cells in intrahepatic CD45+ immune cells of each treatment group (n=6). Data are presented as mean ± SD. One-way AVONA: ns, not significance; *$p < 0.05$.

Materials and methods

Human blood plasma isolation and exosomes collection

Umbilical cord blood, young blood and the old blood were transferred to centrifuge tubes and centrifuged at 1000 g for 10 min to collect the plasma. Subsequently, the plasma underwent a series of low-speed centrifugation steps (1000 g for 10 min, 2,000 g for 10 min) to discard cell debris. Then, the supernatant was centrifuged at 12,000 g (Thermo Scientific™ Nalgene™ Oak Ridge PPCO) for 30 min followed by diluted and ultracentrifugation overnight at 120,000 g. The pelleted exosomes were washed with a large volume of PBS, filtered through a 0.22 μm filter (Merck-Millipore, Darmstadt, Germany), and centrifuged at 120,000 g for 2h, then resuspended in 100 μl PBS. All procedures were performed at 4 °C. Exosomes were stored at -80 °C or used for the downstream experiments. In Addition, parts of exosomes were stored at -4 °C within two days for Transmission Electron Microscope. Samples from three different healthy donors were collected successively to obtain CBP-Exos, YBP-Exos and OBP-Exos, respectively. At least three independent experiments were performed to verify the effects of these exosomes on cultured AML12 hepatocytes and primary hepatocytes.

Characterization of CBP-Exos

The particle size of the exosomes were determined by dynamic light scattering measurements using a ZetaView laser scattering instrument (ParticleMetrix, Germany). The morphology of the exosomes was verified by transmission electron microscopy (JEM-2100Plus, Japan). Western blot was performed to identify the exosomal marker,

anti-tsg101, anti-CD63, and anti-Alix.

CBP-Exos uptake assay

Exosomes were labeled with PKH67 fluorescent dye (Sigma) following the manufacturer's instructions. AML12 hepatocytes or PHs were incubated with labeled exosomes at 37 °C for 6 h and washed with PBS. Then, cells were fixed in 4% PFA for 15 min and washed with PBS for twice. Nuclei were stained with DAPI. The red fluorescent signals were detected by laser confocal microscopy (Nikon A1HD25, Japan).

CBP-Exos internalization

Exosomes were labeled with the CM-DiR membrane dye (AAT Bioquest®, America) following the manufacturer's protocol. Briefly, exosomes were mixed with 1 μmol/L CM-DiR, and incubated for 1 h at 37°C. Excess dye was removed by ultracentrifugation at 100,000 g for 60 min at 4 °C, and the pellets were washed once. The final labeled exosomes were injected into mice by the tail intravenous and then the fluorescence imaging distribution was observed using a Small Animal Optical Imaging System (IVIS Spectrum, Perkin Elmer).

Isolation and identification of mouse primary hepatocytes

Primary hepatocytes (PHs) were isolated as described previously[37]. Briefly, mice were infused with a calcium-free HEPES-phosphate buffer A (Calcium and magnesium-free HBSS containing 0.5 mM EGTA, 25 mM HEPES, pH 7.4) via the vena cava for 3-5 min. After the color of the liver changed to a beige or light brown color, collagenase-

containing buffer B (HBSS with 1 mM magnesium and 1 mM calcium, 25 mM HEPES, 1 mg/ml Liberase) was perfused into the liver. After perfusion for 3-5 min and the liver was excised into ice-cold buffer A. Cells from digested livers were teased out, suspended in Buffer A, filtered through a 70 mm cell strainer, and centrifuged at 50 g for 2 min at 4°C. The pellet was washed with Plating Medium (DMEM with 5% FBS and 1% Penicillin/Streptomycin) twice and then mixed with Percoll to a final concentration of 50% and centrifuged at 200 g for 10 min, 4°C. After removing the supernatant, the hepatocyte pellet was washed once with Plating Medium and then cultured in Williams Medium E containing 1% Glutamine on collagen-coated plates (Gibco, America) and antibiotics. After overnight incubation (16 h), PHs were identified by RT-qPCR analysis of ALB, the uptake and release assay of Indocyanine Green, and Immuno-fluorescence observation of DiI-labeled LDL uptaken.

Cell culture and transfection

Two types of mouse hepatocytes, the AML12 cell line and isolated PHs, were used for in vitro experiments. The AML12 hepatocytes were purchased from the ATCC and cultured in DMEM (Procell, China), and the PHs isolated from normal mice liver and cultured in William's E Culture Medium (Gibco, America). For transfection, AML12 hepatocytes and PHs were transfected with the miRNA mimic or miRNA inhibitor using riboFECT CP Transfection Kit (RiboBio, China) according to the manufacturer's instructions.

Experimental Animals and Treatment Assignment

Female WT C57BL/6 mice were obtained from the Beijing Huafu-Kang Biotechnology Co., Ltd. (China). All mice were kept on a 12 h light/dark cycle and provided ad libitum access to food and water. Mouse ALI was induced by intraperitoneal injection of CCl_4 at a dosage of 0.5 μL/g body weight. Mice were given CBP-Exos 10 μg/g body weight per mouse by intravenous injection at 0 h and 1 d after CCl_4 modeling. The liver fibrosis (LF) model was induced by intraperitoneal injection of CCl_4 twice a week for 6 weeks at a dosage of 0.5 μL/g body weight per mouse and mice were given CBP-Exos by intravenous injection of 10 ug/g body weight CBP-Exos per mouse twice a week for 3 weeks at 4th week of modeling.

miRNA high-throughput sequencing

miRNA high-throughput sequencing experiments were performed by GBI Technology (Beijing, China). Total RNA was extracted from CBP-Exos and OBP-Exos derived from three different donors, respectively, and then sequenced using the MGI2000 high-throughput platform with a sequencing length of SE50. Reads containing low quality, joint contamination, and high content of unknown base N were filtered out after sequencing to ensure the reliability of the results. The analysis of miRNA differential expression was performed on the obtained miRNAs data in the multi-omics data system of Dr.Tom of BGI. Data accessible at NCBI SRA database, assession SRP447247).

The antioxidant and anti-apoptotic effects of CBP-Exos in Vitro

To examine the antioxidant activity of CBP-Exos, AML12 hepatocytes, and PHs were co-cultured with 10 μg/mL CBP-Exos for 24 h and then treated with 200 nm H_2O_2 to

induce oxidative stress. ROS burst and scavenging in cells was assessed by DCFH-DA (beyotime, China) probe and quantified by fluorescent microscope. Cell apoptosis was investigated by TUNEL staining (Beyotime, China) or analyzed by Annexin V-PE/7-AAD Apoptosis Detection Kit (Vazyme, China). Mitochondrial injury and dysfunction regarding as mitochondrial permeability transition pore (mPTP), mitochondrial membrane potential (MMP) after treatments were detected by a Calcein AM (beyotime, China) and AnnexinV/Mito-tracker detection kit (beyotime, China), respectively. Mitochondrial morphology was identified by TEM (JEM-1400FLASH, Japan). Normal or control groups were maintained under standard condition or 200 nm H_2O_2 constantly. In addition, Cell Meter™ Apoptotic and Necrotic Multiplexing Detection Kit I * was used to monitor cell apoptotic, necrotic and healthy cells (AAT Bioquest, America). Cell oxidative damage marker of DNA, γ- Histone H2A family member X (γ-H2AX, abcam) were immunoassayed to evaluate ROS-induced DNA damage.

Dual-luciferase reporter assay

To detect whether Bim is a direct target gene for miR-410-3p, a Bim 3' UTR with the normal miR-410-3p-binding site or a Mut binding site was inserted into the psiCHECK-2 reporter vector. The constructed reporter vectors were co-transfected with the miR-410-3p mimic or its negative control into HEK293T cells and incubated for 48 h. Subsequently, cells were harvested and lysed, and the luciferase activity was measured with a Dual-Luciferase Reporter Gene Assay Kit (LP001, GeneCopoeia, America) following the manufacturer's manual.

RNA isolation and Real-time PCR analysis

Total RNA from liver tissues was extracted for downstream analysis by PrimeScript™ RT reagent Kit (RR047A, Takara, Japan) and TB Green™ Premix Ex Taq™ II (RR820A, Takara, Japan) according to the manufacturer's instructions. Total RNA from the CBP-Exos was extracted with 500 μL TRIzol (Invitrogen, Grand Island, NY) according to instructions supplied by the manufacturer. Subsequently, cDNA was synthesized from RNA using the miDETECT A Track miRNA RT-qPCR Starter Kit (C10712-1, RiboBio, China) and real-time PCR was performed with TB Green™ Premix Ex Taq™ II (RR820A, Takara, Japan). Relative gene expression folding changes were identified with the 2-ΔΔCt method. The sequences of primers used in this study are shown in Table S1.

Liver oxidative damage and apoptosis assessment

The oxidative stress after ALI was evaluated when liver sections were obtained 1 d,2 d,3 d, and 5 d after treatments, and Amplite™ Fluorimetric Malondialdehyde (MDA) Quantitation Kit (AAT Bioquest, America) were used to indicate ROS in the liver. Meanwhile, cardiomyocyte mitochondrial morphology and structure were investigated by TEM. Cell injury and apoptosis were evaluated by tissue cleaved-caspase3 immunostaining and TUNEL staining.

Flow cytometry analysis of immune cell population and cytokine assays

Flow cytometry analysis was applied to detect the collected mononuclear cells isolated from livers in vivo. The livers were harvested, minced, and digested in DMEM medium

containing 200U type I collagenase, 200U type IV collagenase, and 0.1% super nuclease at 37°C for 40–60 min, and the cell suspensions were filtered. Red Blood Cell Lysis Buffer was used to remove erythrocytes and subsequently stained with Fixable viability stain 700 to discriminate between live and dead cells. Finally, the cells were blocked with Fc-Blocker and stained with antibodies. The data were acquired using a NovoCyte flow cytometer (LSR Fortessa, BD, America). Four major immune cytokines levels (TNF-α, IL-6, IL1-β, and IL-10) were determined using ELISA kits following the manufacturer's protocol (Ruixinbio, China).

Immunofluorescence staining

Livers were snap frozen in optimum cutting temperature (O.C.T, Fisher Healthcare) with dry ice. Six mm cryo-sections of tissue sections or cells were cut and fixed with 4% paraformaldehyde for 15 min. Samples were blocked with 5% normal goat serum for 60 min at room temperature. After washing, the samples were incubated with primary antibodies at 4 °C overnight. The secondary antibody was added and subsequently incubated for 1 h at room temperature. Nuclei were stained with DAPI for 10 min at room temperature. Images were observed and acquired on a Laser Confocal Microscope (Nikon A1RMP^{+}, Japan).

Histological analysis and immunohistochemistry

Liver tissues were harvested, immediately fixed with 4% paraformaldehyde, and embedded in paraffin for subsequent use. Deparaffinized liver sections were sectioned at 4-μm thickness for liver histology and stained with red hematoxylin and eosin (H&E)

or sirus red stain (SR). Deparaffinized liver sections were subjected to citric acid buffer (PH6.0) microwave antigen retrieval for immunohistochemistry and then treated with 0.3% H_2O_2 solution to block endogenous peroxidase. After washing, the sections were blocked with non-immune serum and incubated overnight with primary antibodies at 4°C. These sections were then incubated with a chromogenic reagent until the liver sections turned brown. Four to eight independent liver sections were randomly collected using Nikon ECLIPSE E600 and the number of positive cells was quantified using ImageJ software.

Western blot analysis

Cells were harvested in RIPA lysis buffer (Beyotime, Shanghai, China), quantified by a BCA Protein Assay Kit (Thermo, America), separated by 10% SDS-PAGE and transferred to polyvinylidene fluoride membranes (0.45μm, Immobilon-P Transfer Membranes, Merck Millipore. After blocking with 5% skim milk for 2h, the membranes were incubated with primary antibodies at 4 °C overnight. After three times washing with TBST, the membranes were incubated with secondary antibodies for 1 h at room temperature. The chemiluminescent substrate ECL kit (Yamei, Shanghai, China) was used to detect the signal.
